# One‐Step Preparation of Lanthanide‐Doped Polyurea Multicolor Microspheres for Versatile Fluorescent Applications

**DOI:** 10.1002/advs.76091

**Published:** 2026-06-12

**Authors:** Guiyu Zhang, Xiaoxia Yu, Xinhui Wang, Xiang Zheng Kong, Xiangling Gu, Jitao Liu, Shusheng Li, Xiaoli Zhu, Lianbao Cao, Xubao Jiang

**Affiliations:** ^1^ College of Chemistry and Chemical Engineering University of Jinan Jinan China; ^2^ Shandong Cancer Hospital and Institute Shandong First Medical University and Shandong Academy of Medical Sciences Jinan China; ^3^ School of Health and Medicine Dezhou University Dezhou China

**Keywords:** 3D printing, in vivo metabolism, latent fingerprint visualization, Ln^3+^ coordination, multicolor fluorescence, polyurea microspheres, reusable for pollutant detection

## Abstract

Lanthanide‐doped polyurea microspheres (LTP@PUMs) are prepared through a facile one‐step precipitation polymerization, without stabilizer under quiescent condition. The process features simple operation, mild condition and low LTP doping. The size of the microspheres and their fluorescence color are precisely adjustable by tuning experiment conditions. LTP@PUMs are of great interests in several aspects, including multicolor fluorescence, high quantum yield (75.3%), remarkable stability, redispersibility and biocompatibility, owing to good compatibility of LTP with PU, strong hydrogen‐bonding and coordination interactions. These excellent properties allow LTP@PUMs to achieve versatile applications. Taking LTP@PUMs as fluorescent sensor, selective detection for 4‐nitrophenol is shown with high sensitivity (detection limit: 0.076 µM) and good reusability. In latent fingerprint imaging, high‐quality and durable fluorescence images on various substrates are easily obtained. Strong fluorescence is also retained after heating to 260°C, making LTP@PUMs a high‐thermal‐resistant multicolor filler for 3D printing, an interesting property rarely reported. In vivo, LTP@PUMs are demonstrated to accumulate selectively in spleen (71.0%, 2.87 µm) and lungs (90.1%, 10.12 µm), enabling high‐contrast imaging without obvious abnormal behaviors observed after eight months. This work offers, therefore a new strategy for the fabrication of multifunctional fluorescent microspheres, with promising prospects in environmental monitoring, public security, advanced manufacturing, and biomedicine.

## Graphical abstract

Lanthanide‐doped polyurea microspheres are prepared via a facile one‐step precipitation polymerization. They exhibit strong hydrogen‐bonding and coordination interactions with excellent properties, including multicolor emission, high quantum yield (75.3%), remarkable stability, redispersibility, and biocompatibility. These integrated properties enable versatile fluorescence applications in reusable detection of pollutant, latent fingerprint visualization, high‐temperature (260°C) and multicolor 3D printing, in vivo bioimaging, and microplastics tracking.

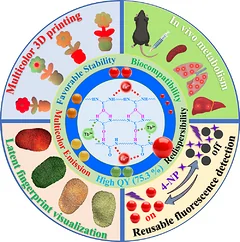

## 1 Introduction

Multicolor fluorescent materials exhibit broad applications in divers fields such as data encryption, chemical sensing, smart displays and bioimaging, thanks to their tunable emission intensity and colors as well as their excellent stimulus responsiveness [1, 2, 3]. Conventional methods for achieving multicolor emission typically rely on introducing luminescent species, such as organic fluorophores [4], fluorescent nanoparticles [5] or lanthanide (Ln) complexes [6], through covalent bonding or physical doping strategies. Ln complexes have garnered, in particular, significant interest as building blocks for high‐performance fluorescent materials, owing to their distinctive f–f electronic transition, which confers a narrow emission band, superior color purity, extended fluorescence lifetime, and large Stokes shift [2, 3, 6]. However, Ln complexes generally exist in powder form, making it difficult for their processing. They are also prone to aggregation and poorly stable. To address these issues, Ln‐doped polymer composites have been developed, through combination of the high fluorescent performance of Ln^3+^ with selective base‐polymers, which are usually of structural robustness, processability, and easy functionalization [7, 8, 9, 10].

For the fabrication of Ln‐doped polymer composites, a very crucial step involves incorporation Ln complexes into polymer matrices [11, 12, 13, 14, 15, 16, 17], with the characteristic emission profiles of Ln^3+^ well kept in the outcome hybrid materials, and so are the desired properties of the polymer microspheres, such as mechanical stability, small size (micro‐/nanoscale), high specific surface area and easy functionalization, making the composite highly potential for uses in data encryption, chemical sensing and biomedicine [11]. Therefore, the fabrication of Ln‐polymer composite microspheres with tunable fluorescence is of significant scientific and practical interests. Common methods for the incorporation of Ln complexes into polymer microspheres include polymerization (e.g., emulsion and dispersion polymerization) in the presence of Ln complexes [12, 13, 14], and treatments of a prefabricated polymer using Ln complexes (e.g., swelling‐shrinking method and oil‐in‐water emulsion) [15, 16, 17]. These approaches involve, quite often, complex procedures and uses of specific surfactants or organic solvents, limiting their practical applications, in particular in biomedical fields. In sharp contrast, precipitation polymerization, without need for any emulsifiers or stabilizers, provides a straightforward route to obtain clean‐surfaced polymer microspheres [11, 18, 19, 20, 21, 22]. Currently common strategies for endowing fluorescence with polymers prepared through precipitation polymerization mainly include: use of functional monomers bearing emissive moieties (e.g., BODIPY, pyrene, tetraphenylethylene) in the polymerizing, [23, 24, 25] and microsphere post‐modification with fluorophores (e.g., TAMRA‐cadaverine, spiropyran derivatives) [26, 27]. These methods involve not only multi‐step procedures, but also often rely on costly/complex fluorophores, and yield only single‐color emission in generally [23, 24, 25, 26, 27]. To date, few studies have been reported with regard to the preparation of Ln‐doped multicolor fluorescent polymer microspheres via precipitation polymerization.

In a previous work, highly uniform polyurea microspheres (PUMs) were obtained through precipitation polymerization under quiescent conditions, using isophorone diisocyanate (IPDI) as the sole monomer without any stabilizer and initiator [28, 29, 30, 31]. We herein report a one‐step preparation of Ln(TTA)_3_Phen (LTP, Ln = Eu, Tb) doped polyurea microspheres (LTP@PUMs) with multicolor fluorescence. Compared with existing research, the present process is additive‐free, conducted under quiescent conditions, and uses low LTP doping (≤0.5 wt.%). Its operational simplicity and benign reaction parameters significantly reduce both process complexity and cost. Moreover, the size (2–10 µm) and emission colors (red, orange, yellow, green) of the LTP@PUMs can be simultaneously controlled through experimental condition. LTP@PUMs thus prepared exhibit high fluorescence quantum yield (up to 75.3%), good redispersibility, robust stability and biocompatibility, enabling LTP@PUMs to achieve excellent performance across multiple application fields. Systematic investigations confirm their application as fluorescent sensors for 4‐nitrophenol (4‐NP) detection with high sensitivity and selectivity. In addition, their diverse applications are also demonstrated, including the use in latent fingerprint (LFP) visualization, 3D printing as fluorescent additives, and in vivo bioimaging.

## 2 Results and Discussion

### 2.1 Preparation of LTP@PUMs

**FIGURE 1:**
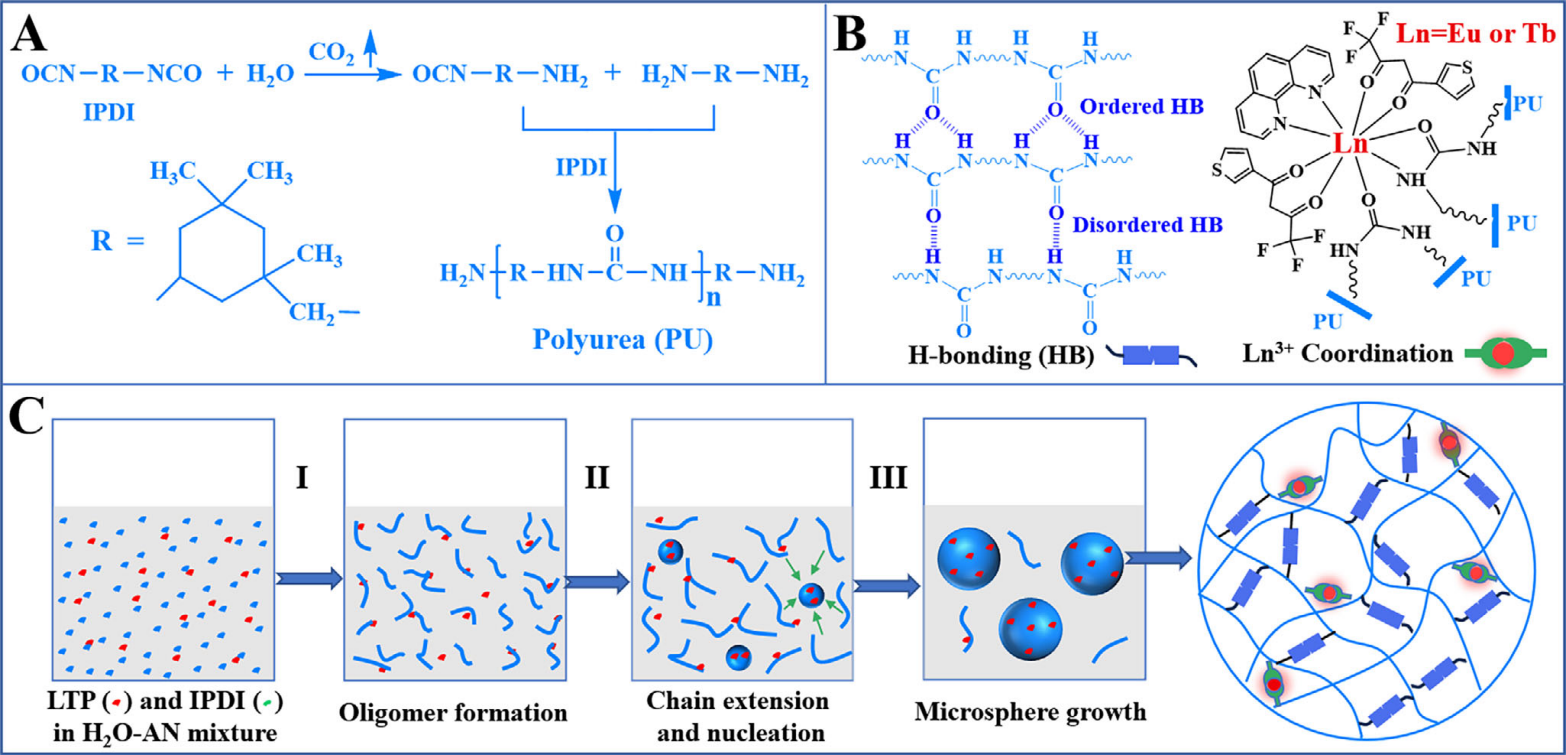
(A) Chemical reactions in PU formation via the reaction of IPDI with water; (B) Illustration of HB and Ln^3+^ coordination in LTP@PUM; (C) Schematic of LTP@PUMs formation process.

Polyurea (PU) is formed by reacting water with the isocyanate (NCO) groups of IPDI, producing primary amines via a proton‐transfer process accompanied by CO_2_ release [28, 29, 30, 31]. These in situ formed primary amines further react with NCO groups, leading to PU formation through step‐growth polymerization (Figure 1A). The high density of urea linkages in PU results in strong hydrogen‐bonding (HB) within the matrix (Figure 1B) [29]. Uniform PUMs are easily obtained using this process through the polymerization in mixed solvents of water with acetone, acetonitrile (AN) or DMF [28, 29, 30, 31]. The present work focuses on the preparation of LTP@PUMs in H_2_O/AN by reacting IPDI with water in the presence of Ln complex (LTP), including Eu(TTA)_3_Phen (ETP) and Tb(TTA)_3_Phen (TTP). It is to point out that none of the LTPs takes part in the IPDI‐water reaction, because they do not possess the reactive groups required in PU formation (Figure 1B and Figure S1). Nevertheless, it is reported that Ln^3+^ are capable of coordinating with carbonyl and amide groups [8, 10, 32, 33, 34, 35]. This constitutes the base mechanism of this work, i.e. incorporation of LTP into PUM through the coordination between PU urea groups and Ln^3+^ (Figure 1B), which will be presented in the following sections. The formation of LTP@PUMs proceeds without emulsifiers or stabilizers. At start, a homogeneous transparent solution is obtained, the characteristic of this precipitation polymerization. Based on the mechanism, [31, 36, 37] the process can be divided into three stages (Figure 1C): (I) IPDI reacts with H_2_O to form PU oligomers, which can coordinate with Ln^3+^ via urea groups; (II) the oligomer chains grow in length and eventually precipitate out upon reaching a critical chain length due to decreased solubility, yielding primary particles; (III) the primary particles continue to grow by adsorbing PU oligomers and their Ln complexes, or through reactions between the terminal groups of oligomers and the surface functional groups on the microspheres.

**FIGURE 2:**
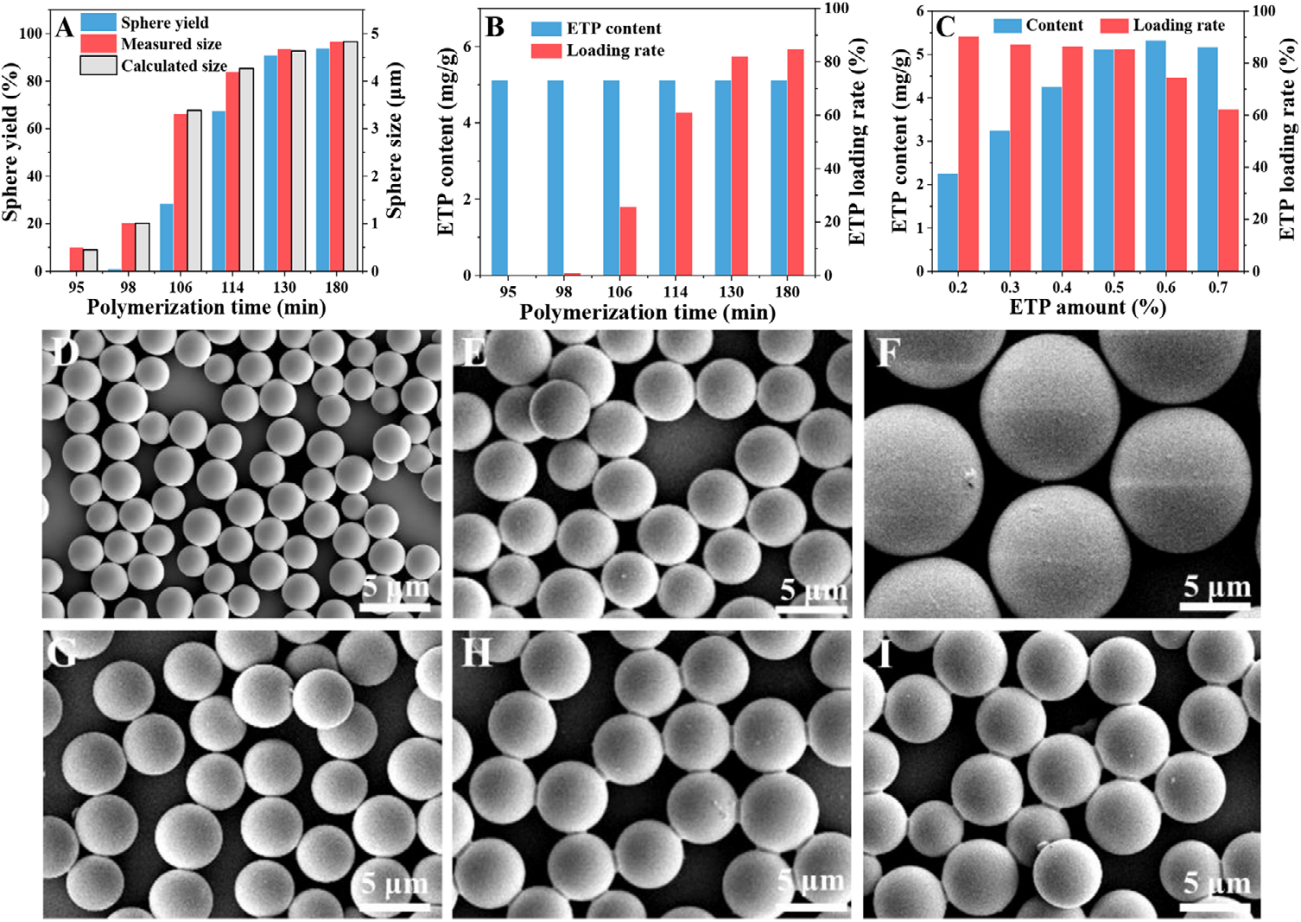
(A) Sphere yield, measured and calculated sphere size of ETP@PUMs obtained at different polymerization time; (B, C) ETP content (mg/g) and loading rate (%) in resulting microspheres prepared at different polymerization time (B) and ETP amount (C, 180 min); (D–I) SEM images of LTP@PUMs prepared under different conditions: (D–F) ETP@PUMs prepared at 30°C (D), 50°C  (E) and 70°C (F), respectively; (G) TTP@PUM prepared at 50°C; (H, I) ETP&TTP@PUMs obtained at 50°C with ETP/TTP mass ratios of 1/15 and 1/21, respectively. All samples were prepared with 8.0% IPDI and 0.5% LTP.

A transparent solution is obtained when IPDI and ETP are added to the H_2_O/AN mixture (20/80 by mass). As the polymerization proceeds, the mixture exhibits increasing turbidity, indicating the formation of primary particles. The turbidity time for pure PUM was about 109 min, whereas for ETP@PUM (with both ETP and IPDI added), it occurred at about 95 min (Table S1), suggesting that ETP incorporation accelerates particle nucleation. In the preparation of ETP@PUM, samples were taken at different time intervals, rapidly quenched in liquid nitrogen to terminate the polymerization, and then freeze‐dried. After repeated washing, centrifugation and drying, sphere yield and size were determined (Figure 2A and Figure S2), which showed that the yield and the size of ETP@PUM were increased with polymerization time, and reached constant at 180 min, indicating the completion of the process. Furthermore, by assuming a constant sphere number throughout the process, theoretical sphere sizes at each sampling time were calculated, based on the yield and the size of the microspheres in the previous sampling [31]. The results show good agreement between the measured and calculated sizes (Figure 2A), suggesting that the sphere number remained stable throughout the process. The final size of ETP@PUM was 4.83 µm, after 180 min of the polymerization, which was smaller than that of pure PUM (7.13 µm, Table S1). This size reduction is attributed to coordination cross‐linking between Eu^3+^ and PU urea groups, which promotes earlier precipitation of PU chains, accelerates particle formation, increases nucleation of the primary particles, leading to a smaller final size therefore. ETP content (mg/g) in ETP@PUM was determined by ICP‐OES, and ETP loading rate (%, relative to total ETP added) was calculated accordingly. The ETP content on the microspheres was around 5.12 mg/g, practically constant throughout the whole process, whereas ETP loading rate increased gradually with polymerization time, stabilizing at 85.40% at 180 min and remained unchanged thereafter (Figure 2B, Table S1). These results suggest that sphere growth proceeds via migration of PU‐Eu^3+^ complexes to the surface of existing microspheres, giving a uniform distribution of ETP within ETP@PUM.

To demonstrate the easy adjustments for sphere size, LTP types, and content, a series of experiments were conducted by varying IPDI loading, LTP amount, polymerization temperature, and ETP/TTP mass ratio, including ETP@PUM (Tables S1–S3), TTP@PUM (Table S4), and composite ETP&TTP@PUM (Table S5). The morphologies of the final samples were characterized by SEM (Figures S3–S5). Results show that ETP@PUM prepared with an ETP amount below 0.6% exhibited uniform sphere size, whereas a higher ETP amount led to a broader size distribution (Figure S3). As the ETP amount increased from 0.2% to 0.6%, sphere size decreased from 5.21 to 4.76 µm, while ETP content in ETP@PUM increased from 2.26 to 5.33 mg/g, and its loading rate decreased from 90.21% to 74.49% (Figure 2C, Table S1), reflecting a lower incorporation efficiency of ETP into the microspheres at a higher ETP loading. In comparison to these results, variation in IPDI loading and the polymerization temperature showed limited effects on ETP content and loading rate in ETP@PUM, although a pronounced influence on sphere size was well seen (Tables S2 and S3, Figure S4). In particular, sphere size decreased markedly from 10.12  to 2.87 µm as the temperature was raised from 30°C to 70°C (Figure 2D–F, Table S3). Additionally, uniform microspheres of TTP@PUM (Figure 2G and Figure S5, Table S4) and ETP&TTP@PUM (Figure 2H,I, Table S5) were also prepared using varying amount of TTP (0.2%–0.6%), and ETP/TTP mixtures at different mass ratios (1/13–1/25) with total LTP (ETP+TTP) amount fixed at 0.5%. Practically the same profiles as for ETP@PUM were observed, for sphere size and yield, LTP content and loading rate, indicating that the type of Ln complex (ETP, TTP) has minimal influence on the formation of the composite microspheres.

### 2.2 Characterization of LTP@PUMs

**FIGURE 3:**
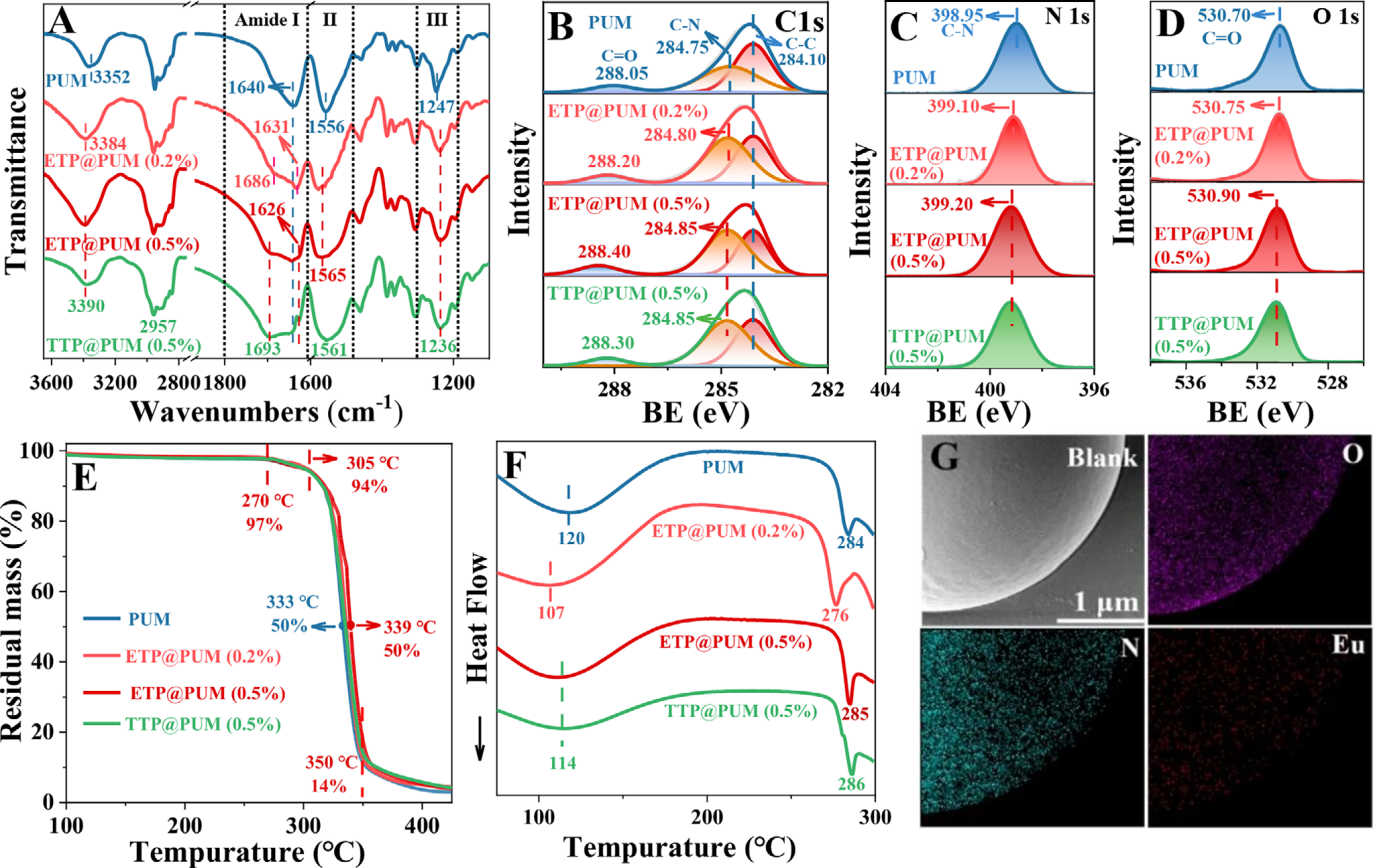
(A) FTIR spectra of PUM, ETP@PUM (0.2% and 0.5% ETP) and TTP@PUM; (B‐D) Deconvolved XPS spectra of the corresponding samples in the (B) C 1s, (C) N 1s and (D) O 1s regions; (E) DSC and (F) TGA curves of the same samples; (G) EDS elemental mappings of ETP@PUM.

Chemical structures and interactions in PUM and LTP@PUMs were first characterized using FTIR. The strong absorption peak at 2247 cm‐^1^, assigned to NCO stretching vibration of IPDI [29, 30, 31], completely disappeared in PUM and LTP@PUMs (Figure S6), indicating complete NCO consumption. Meanwhile, both spectra of PUM and LTP@PUMs exhibited characteristic urea bands, including N─H stretching in 3600–3200 cm^−1^ region, C = O stretching (amide I) near 1650 cm^−1^, N─H bending (amide II) at about 1560 cm^−1^, and C─N stretching coupled with N─H bending (amide III) around 1240 cm^−1^, confirming the formation of urea linkages [29, 38, 39]. The peak at 2957 cm^−1^, attributed to C‐H stretching vibrations from IPDI‐derived units, was also observed. HB characteristics were analyzed using the N─H stretching and C = O amide I regions. Typically, free urea groups (non‐hydrogen‐bonded) display N─H and C = O vibrations above 3460 and 1710 cm^−1^, respectively. HB urea species can be categorized into two types (Figure 1B): [40, 41, 42] (I) ordered HB, involving bidentate interactions between one C = O and two N─H groups, which promotes chain ordering and appears at lower frequency (N─H: <3350 cm^−1^; C = O: <1650 cm^−1^); (II) disordered HB, involving single bond between C = O and one N─H group, yielding intermediate frequency (N─H: 3350–3460 cm^−1^; C = O: 1650–1710 cm^−1^). As seen in Figure 3A, pure PUM showed N─H stretching peak at 3352 cm^−1^ (bandwidth: 3200‐3500 cm^−1^) and C = O stretching peak at 1640 cm^−1^ (1610–1760 cm^−1^). These peaks located at the boundary between ordered and disordered HB regimes, indicating the coexistence of both HB types in PUM, with a minimal population of free urea groups.

The incorporation of 0.2% ETP induced significant changes in the spectra of ETP@PUM (Figure 3A). Compared with PUM, the N─H stretching vibration shifted from 3352 to 3384 cm^−1^. While the primary amide I band remained at 1640 cm^−1^, two new shoulders emerged: a high‐frequency shoulder at 1686 cm^−1^ (blue‐shifted) and a low‐frequency one at 1631 cm^−1^ (red‐shifted), resulting in noticeable broadening of the amide I band. In addition, the amide II band blue‐shifted from 1556 to 1565 cm^−1^, whereas amide III band red‐shifted from 1247 to 1236 cm^−1^. These alterations reflect a substantial disruption of the native HB network in PUM, attributable to the coordination of Eu^3+^ with O and N atoms in the urea groups [8, 10]. HB and Eu^3+^ coordination could produce antagonistic effects in FTIR spectra of ETP@PUM: a decrease in HB order enhances the force constants of N─H and C = O, leading to blue‐shifted peaks, [43, 44] which is consistent with the shifts observed in the N─H stretching (3384 cm^−1^) and amide I&II (1686 and 1565 cm^−1^) bands (Figure 3A). Conversely, Eu^3+^ coordination reduces electron density on O and N atoms, lowering the force constants of C = O and N─H bonds and resulting in a red‐shifting, [8, 10] as evidenced by the low‐frequency amide I shoulder (1631 cm^−1^) and the red‐shift of amide III band (1236 cm^−1^). These results clearly demonstrate that Eu^3+^ incorporation disrupts both ordered and disordered HB networks in PU, to create new coordination interactions. With ETP amount increased to 0.5%, these spectral variations became more pronounced (Figure 3A): the N─H stretching further blue‐shifted to 3390 cm^−1^, the high‐frequency amide I shoulder moved to 1693 cm^−1^, the low‐frequency one red‐shifted to 1626 cm^−1^, and the amide II&III bands exhibited increased broadening. Furthermore, FTIR analysis was also performed for TTP@PUM and ETP&TTP@PUM prepared with the same LTP amount (0.5%, Figure 3A and Figure S6). The spectral profiles showed no notable difference from those of ETP@PUM, an indication that different LTPs (ETP and TTP) have the similar interacting with PU matrix, i.e. the proposed competition interactions between HB and Ln^3+^ coordination.

XPS was then employed to probe the coordination in LTP@PUMs. The C 1s spectrum of PUM was deconvoluted into three components: C–C/C–H at 284.10 eV, urea C–N at 284.75 eV, and C = O at 288.05 eV, with N 1s and O 1s binding energies (BE) observed at 398.95 and 530.70 eV (Figure 3B–D), respectively [10, 43]. Systematic BE shifts were observed upon ETP incorporation. At 0.2% ETP amount, the peak of C 1s in C─N shifted to 284.80 eV while N 1s increased to 399.10 eV. At 0.5% ETP, these peaks changed to 284.85 and 399.20 eV, respectively (Figure 3B,C). Meanwhile, the peak of C 1s in C = O shifted to 288.20 (0.2% ETP) and 288.40 eV (0.5% ETP), and the O 1s peaks shifted to 530.75 and 530.90 eV (Figure 3B,D), respectively. These consistent BE changes indicate electron density decrease on N and O sites. HB dissociation and Eu^3+^ coordination exert opposite electronic effects: while HB dissociation increases electron density on O and N atoms, Eu^3+^ coordination with these two atoms produces a net electron decrease on these atoms along with an increase on Eu^3+^ center [10, 33, 43, 44]. The predominant BE increases in C─N and C = O bonds suggest that the electron‐donating of N and O atoms in their Eu^3+^ coordination outpaces that of N and O atoms in HB. Moreover, the Eu 3d_3/2_ and 3d_5/2_ peaks shifted from 1164.3 and 1134.6 eV, in pure ETP, to 1165.6 and 1135.9 eV, in ETP@PUM, respectively (Figure S7), suggesting a likely electron density decrease on Eu^3+^ center due to partial ligand exchange from TTA/Phen to urea groups. The enhanced BE reflects also the stronger electron‐withdrawing character of urea groups, which increases the ionic nature of the Eu‐ligand bonds. XPS spectra of TTP@PUM and ETP&TTP@PUM (Figure 3B–D, Figures S7 and S8), prepared under identical conditions, showed nearly the same peak positions to those of ETP@PUM. In addition, the XPS spectra of Tb^3+^ (Figure S7C) showed a clear shift in the Tb 3d peaks from 1276.5/1242.4 eV in pure TTP to 1277.7/1243.6 eV in TTP@PUM, also indicating a partial ligand exchange from TTA or Phen to urea groups. These results clearly demonstrated that Ln^3+^ forms charge‐transfer coordination bonds with urea groups, disrupting their HB networks and facilitating direct Ln^3+^‐N/O bonds, in good agreement with the results from FTIR analysis.

Thermal behavior and phase transitions were then evaluated using TGA and DSC for PUM and LTP@PUMs. TGA results (Figure 3E) showed that PUM and LTP@PUMs exhibit high thermal stability, with weight losses of only about 3% and 6% at 270°C and 305°C, respectively. The incorporation of LTP improved the thermal stability, as indicated by an increase in the temperature of 50% mass loss, from 333°C for PUM to 339°C for ETP@PUM. At 350°C, the residual mass was 10.3% for PUM, while this value became 10.9% for ETP@PUM with 0.2% ETP incorporation, and increased further to 15.7% for ETP@PUM with 0.5% ETP. This trend was well kept till the very end of the TGA test at 425°C. DSC analysis (Figure 3F) revealed two endothermic events for PUM: one at 120°C associated with the annealing process [41, 45], the other at 284°C attributed to the melting of microcrystalline regions [45, 46, 47]. It is noteworthy that thermal decomposition of PUM started at around 270°C (Figure 3E), ahead of the microcrystalline melting (284°C). This behavior originates from the high HB density of PUM. Upon the incorporation of 0.2% ETP, the annealing temperature decreased to 107°C (from 120°C for PUM), and the melting peak decreased, from 284°C for PUM to 276°C (Figure 3F), most likely due to the disruption of ordered HB network, which facilitates molecular rearrangement at lower energy. By increasing the ETP amount to 0.5%, the annealing temperature raised further to 114°C, and the melting to 285°C (Figure 3F), respectively, owing to most likely an enhanced coordination‐based cross‐linking, which will restrict chain mobility at certain extent. Similar thermal behavior was also observed for TTP@PUM and ETP&TTP@PUM (Figure 3F and Figure S9), underscoring the general influence of Ln^3+^ coordination on PU thermal behavior.

XRD was also employed to probe the microstructures of PUM and LTP@PUMs (Figure S10). PUM pattern displayed a broad diffraction peak centered at 2θ = 14.3°, and this peak slightly shifted to 14.7° in LTP@PUMs, suggesting a structural adjustment upon LTP incorporation. The broad profiles of the diffraction peaks, for all the samples regardless of incorporated LTP types, confirms that PU matrix is predominantly amorphous, comprising only a quite low amount of ordered microcrystalline domains, or microdomains [46]. The low crystallinity is believed owing to the chain asymmetry imparted by IPDI's cyclohexane ring and its substituents, which hamper long‐range ordering despite HB presence. However, the melting endotherm (276°C–286°C) observed by DSC affirms that even these minimally ordered microdomains contribute discernibly to the thermal behavior of PUM and LTP@PUMs (Figure 3F). Moreover, elemental mapping via EDS was conducted to visualize the spatial distribution of Eu and Tb elements within ETP@PUM and TTP@PUM (Figure 3G and Figure S11). Due to the low Eu and Tb contents (<0.5%), their signals were much weaker than those of the major elements (e.g., C, O, N). However, EDS mapping suggests smooth and homogeneous distributions for Eu and Tb, without even slight localization, aggregation or phase separation.

### 2.3 Fluorescence Behavior of LTP@PUMs

**FIGURE 4:**
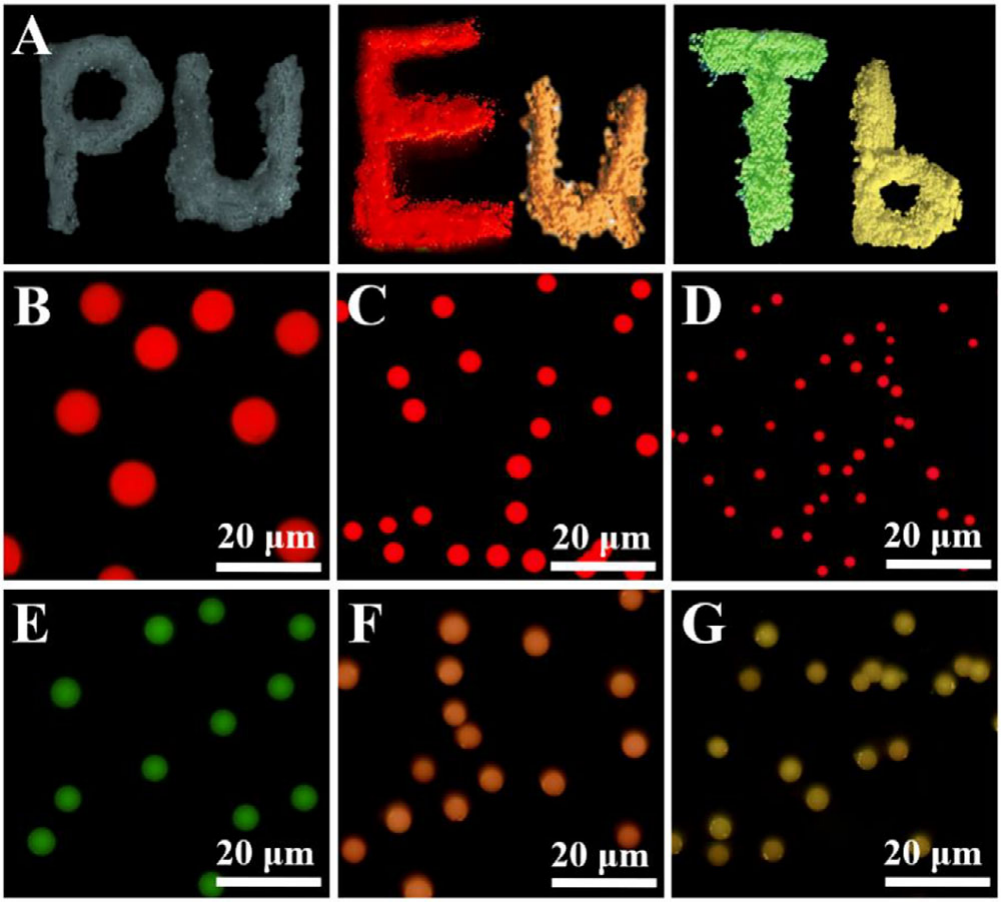
(A) Photographs of different microsphere powders under 365  nm UV irradiation (Label “PU” for PUM; “E” in red: ETP@PUM; “T” in green: TTP@PUM; Label “u” in orange and “b” in yellow: ETP&TTP@PUM prepared with mass ratios of 1/15 and 1/21); (B–G) FM images of LTP@PUMs obtained with total LTP of 0.5% under different conditions (excitation wavelength: 320–400  nm): ETP@PUM prepared at 30°C (B), 50°C (C), and 70°C (D); TTP@PUM prepared at 50°C (E); ETP&TTP@PUM obtained at 50°C with ETP/TTP mass ratios of 1/15 (F) and 1/21 (G).

Under 365 nm UV irradiation, pure PUM powder exhibits only weak bluish fluorescence (labeled “PU” in Figure 4A), whereas LTP incorporation makes radical changes for the emission, i.e., bright red emission of ETP@PUM and green emission of TTP@PUM (“E” and “T” in Figure 4A). The incorporation in PUM was also done with both ETP and TTP, with ETP/TTP mass ratio of 1/15 at the same amount (0.5%) as for ETP in ETP@PUM. It is interesting to see that, the resulting ETP&TTP@PUM displays orange emission (“u” in Figure 4A), and this emission shifts to yellow (“b” in Figure 4A) with the binary incorporation done with ETP/TTP ratio changed to 1/21. Fluorescence microscopy (FM) images provide a more detailed visualization of the emission behavior of LTP@PUMs (Figure 4B–G). Specifically, ETP@PUMs prepared at different polymerization temperatures (30°C, 50°C, and 70°C) all exhibit red emission (Figure 4B–D), with a clear decrease in sphere size with the temperature increases. Whereas TTP@PUM, prepared at 50°C, shows green emission (Figure 4E), while the binary incorporated ETP&TTP@PUM exhibit orange and yellow emission with ETP/TTP ratio of 1/15 and 1/21 (Figure 4F,G), respectively. The emission color of ETP&TTP@PUM is the combination of the red emission from Eu^3+^ and the green emission from Tb^3+^. Therefore, fluorescent microspheres with tunable size and emission color are efficiently prepared by simply adjusting polymerization temperature, the type and the ratio of Ln complexes.

**FIGURE 5:**
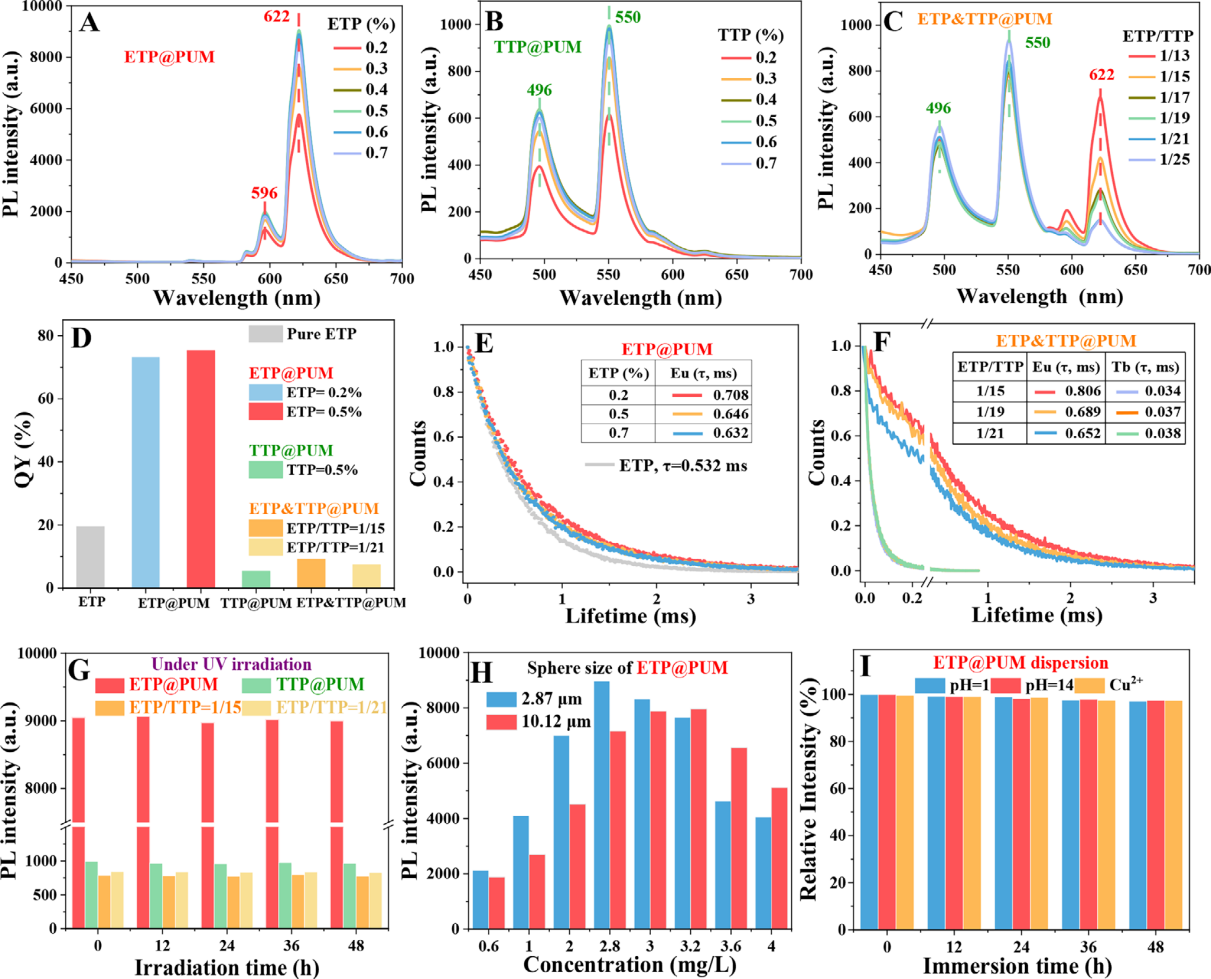
Fluorescence emissions under 𝜆_ex_ = 360 nm (𝜆_em_ = 622 nm for Eu^3+^, and 550 nm for Tb^3+^): (A–C) Spectra of solid powders: (A) ETP@PUM, (B) TTP@PUM and (C) ETP&TTP@PUM, prepared with varying ETP, TTP amount and ETP/TTP ratios; (D) QY of ETP and various LTP@PUMs; (E) Fluorescence decay curves of Eu^3+^ emission in pure ETP and ETP@PUM; (F) Fluorescence decay of Eu^3+^ and Tb^3+^ emission in ETP&TTP@PUM; (G) Emission intensity of different LTP@PUM powders under 365 nm UV light for different irradiation time; (H) Emission intensity of ETP@PUM dispersions (2.87 and 10.12 µm) as function of microsphere concentration; (I) Relative emission intensity of ETP@PUM immersed in a Cu^2+^ solution (20 mM), and in aqueous solutions of pH  1 and 14 for different time.

The emission behavior was examined by fluorescence spectroscopy for ETP, PUM, and ETP@PUMs powders. ETP exhibits four sharp emission peaks, at 582, 596, 622, and 702  nm (Figure S12A), corresponding to Eu^3+ 5^D_0_→^7^F_J_ (J = 0, 1, 2 and 4) transitions [10, 48, 49]. In contrast, PUM displays only weak emission in the blue region, with an intensity about 10% of ETP emission under the same excitation wavelength (𝜆_ex_, Figure S12B). The characteristic peak of Eu^3+^ at 622  nm is of the highest intensity in the emission spectra of ETP@PUMs (Figure 5A and Figures S13–S15), in accord with the strong red fluorescence shown in Figure 4B–D. The peak position is unchanged under different 𝜆_ex_, and a maximum intensity is observed with 𝜆_ex_ = 360  nm (Figure 5A and Figure S13). As the ETP amount increased, the emission intensity increased first, reaching a maximum at ETP amount of 0.5%, followed by a slight decline in intensity (Figure 5A) with further increases in ETP amount, likely due to aggregation‐caused quenching (ACQ) because of the concentration‐induced ETP aggregation [10, 50]. It is found that, ETP@PUM emission exhibited a slight decrease with increasing IPDI concentration and polymerization temperature (Figures S14 and S15) while ETP amount was kept always at 0.5% of the IPDI amount. As indicated in Tables S2 and S3, actual ETP content in ETP@PUMs slightly decreased under these conditions, reducing the chromophore density and thus the observed decrease in emission intensity. Therefore, the emission intensity is effectively and easily tuned by the incorporated ETP content in ETP@PUMs.

Pure TTP complex is of very feeble fluorescence under irradiation of 360  nm (Figure S12A), due to the mismatch between the triplet energy level of the ligand TTA and the Tb^3+^ energy level (^5^D_4_), leading to ineffective energy transfer from TTA to Tb^3+^ center [51, 52]. In contrast, emission spectra of TTP@PUM displays characteristic emission peaks of Tb^3+^ at 496, 550, 585, and 624  nm (Figure 5B and Figure S16), owing to its ^5^D_4_→^7^F_K_ (K = 6‐3) transitions [5, 33, 49]. The peak at 550  nm (^5^D_4_→^7^F_5_) is of the highest intensity and is responsible for the green fluorescence. The emission peaks remain unchanged under different 𝜆_ex_ and TTP content, with maximum intensity obtained under 𝜆_ex_ = 360 nm. The fluorescence profiles of TTP@PUM versus TTP, together with the Tb^3+^ XPS data (Figure S7C), indicate that Tb^3+^ coordination with urea is in fact a process to replace TTA ligand in coordination with Tb^3+^, by O and N atoms in the urea group. Prior to this ligand switch, Tb^3+^ was coordinated with TTA with its emission practically suppressed; and Tb^3+^ emission was effectively reactivated by switching its coordination with TTA to that with the urea group. In addition, PU chains may act as new sensitizers, activating the f–f transition of Tb^3+^ through an energy transfer [49]. As the TTP amount increases, the emission intensity at 550  nm first increases, reaching a maximum at 0.5% TTP (Figure 5B), followed by a decrease in emission intensity with further TTP increase, likely owing to the ACQ effect [10, 50]. The transition from “non‐fluorescent” to “fluorescent” state, together with the clear dependence on TTP content in the composite, provide strong evidence that the coordination between PU and Tb^3+^ is the fundamental mechanism for Tb^3+^ fluorescence in TTP@PUM.

The emission spectra of ETP&TTP@PUM exhibit characteristic emission peaks of both Eu^3+^ and Tb^3+^, well seen as the most intense emission at 622  nm for Eu^3+^ and that at 550 nm for Tb^3+^, both observed under 𝜆_ex_ = 360 nm (Figure 5C and Figure S17). These emission intensities can be effectively regulated by changing the ratio of ETP/TTP. With the ratio increased, the intensity at 622 nm (of Eu^3+^) gradually increases, and that at 550 nm (of Tb^3+^) decreases (Figure 5C), allowing easy adjustment for the emission color across the range from green (TTP only) to yellow (1/21), orange (1/15), and red (ETP only, Figure 4).

Fluorescence quantum yields (QYs) were determined for ETP, PUM, and LTP@PUMs (Figure 5D). A QY of 19.5% is detected for pure ETP. Once incorporated into PU matrix, significant increases are seen for the QY: 73.2% for the ETP@PUM with ETP of 0.2%, and 75.3% with ETP of 0.5%. The high QY of ETP@PUM are partly related to efficient energy transfer in the system [10, 49]. To provide evidence for this energy transfer in ETP@PUM, UV–vis absorbance of PU and the fluorescence excitation of ETP and EuCl_3_ were tested (Figure S18A). The results show that PUM exhibits strong absorption in the range of 200–500 nm, while the excitation spectra of ETP and EuCl_3_, monitored at 622 nm, display broad bands within the same region (200–500 nm). The strong absorption covers the entire excitation range of Eu^3+^, with a substantial spectral overlap between them, indicating efficient energy transfer. Furthermore, other key factors also play an important role in achieving remarkable QY enhancements: (I) The abundant HB interaction provide high structural rigidity to PU chains, effectively suppressing non‐radiative relaxation processes such as intramolecular rotation and ETP vibration, thereby reducing energy loss [49, 53, 54]. (II) The uniform ETP dispersion and its good compatibility with PU matrix, as confirmed by EDS mapping, would delay or largely attenuate the ACQ effect [10, 50]. (III) The strong coordination between Eu^3+^ and urea groups, as shown by FTIR and XPS analyses, enhance further the emission efficiency through energy transfer from PU to Eu^3+^ [7, 10, 49]. In steep contrast to ETP@PUMs, a much lower QY (5.4%) was detected for TTP@PUM. It is to point out that, pure TTP is nearly non‐emissive due to energy level mismatch. This low QY is seen as a substantial ramp‐up, a solid support for the strong coordination and energy transfer between PU and Tb^3+^. For the co‐doped systems with ETP/TTP mass ratios of 1/15 and 1/21, the overall QY was 9.2% and 7.4% for the samples (Figure 5D), respectively, which indicates that the QY is determined by the relatively low emissive Tb^3+^, as expected because the co‐doping was done excessively by Tb^3+^.

Fluorescence lifetime (τ) measurement was also performed to investigate the excited‐state decay in PUM and LTP@PUMs. When excited at 360 nm, PUM exhibited weak emission at 400 nm with a lifetime of 3.960 ns (Figure S18B), while PU emission in ETP@PUM was fully quenched off with no lifetime data detectable. Eu^3+^ lifetime in pure ETP (622 nm) is 0.532 ms, which increased to >0.632 ms in ETP@PUM (Figure 5E). These results demonstrates a distinct energy transfer from PU to Eu^3+^ [10, 53, 54]. Eu^3+^ lifetime in ETP@PUM decrease gradually from 0.708 to 0.632 ms as ETP amount increased from 0.2% to 0.7%. This systematic reduction with ETP amount is caused by reduced distance between Eu^3+^ because of its content increase, which enhances intermolecular non‐radiative energy transfer and accelerates the decay of the excited state [7, 10, 50]. A similar trend was also observed in TTP@PUM (Figure S18C), and a much shorter lifetime (0.032–0.041 ms) of Tb^3+^ was seen in comparison to those of Eu^3+^ in ETP@PUM, due to the energy level mismatch between Tb^3+^ and the residual ligand TTA. Eu^3+^ lifetime increased slightly to 0.652 ms for the co‐doped ETP&TTP@PUM prepared with ETP/TTP ratio of 1/21(Figure 5F), from 0.646 ms for ETP@PUM. By further increasing the ETP content to a mass ratio of 1/15 in the co‐doped composite, a more pronounced extension of Eu^3+^ lifetime to 0.806 ms was clearly observed. It is reported that the higher excitation energy level of Tb^3+^ enables it to act as an efficient energy donor to Eu^3+^ [10, 49], i.e., an energy transfer, from Tb^3+^ donors to Eu^3+^ acceptors is assumed to occur, extending therefore the emission lifetime of Eu^3+^. In contrast, Tb^3+^ lifetime remained almost unchanged within the narrow range (0.034‐0.038 ms), which is attributed to the substantially higher Tb^3+^ doping (at least 15‐fold of Eu^3+^), making them less sensitive to variations in the local environment.

To assess the thermal stability, LTP@PUM powder was heated to and kept at 85°C for up to 48 h, and the emission was recorded at different time intervals (Figure S19). Another set of stability tests against UV irradiation was also done by keeping the samples under irradiation with 365 nm UV light for up to 48 h at room temperature, and the emission was recorded every 12 h (Figure 5G). The results indicate that, in the two sets of tests, the emission intensity was practically unchanged for all samples, regardless of their incorporating partners, the heating and irradiation time. These results demonstrate high stability and long‐term operational reliability, which we believe are attributed to the strong HB and coordination within LTP@PUM (Figure 3). The eventually structural vibration and non‐radiative relaxation of LTP complexes are largely attenuated or fully suppressed even under harsh thermal stress or UV irradiation, preventing therefore the quenching of their fluorescence.

LTP@PUMs, i.e., ETP@PUM and TTP@PUM, were then dispersed each in water to form aqueous suspension (3 mg/mL), and the emission behavior and stability were studied for both suspensions. Practically identical emission spectra were obtained for the dispersions as for the solid powders, and the emission intensity remained virtually unchanged within 2 h of still standing at room temperature (Figure S20). All fluorescence spectra were recorded using an F‐7000 spectrophotometer with a scan time of less than 2 min per spectrum. Within this timeframe, the fluorescence signals remained stable, indicating that the effect of the potential microsphere sedimentation during measurement on the emission intensity is negligible. The concentration‐dependent emission behavior was examined using ETP@PUMs of 2.87 and 10.12 µm (Figure 5H). For ETP@PUM of 2.87 µm, its emission intensity increased with concentration, reaching a maximum at 2.8 mg/mL, followed by a decrease thereafter. At low concentrations, the intensity enhancement correlates with the increasing number of fluorescent centers. However, at elevated concentrations, the light transmittance of the dispersion decreased (Figure S21), and the light scattering intensified, which makes the penetration of the excitation light hampered at some extent, leading to lower apparent fluorescence intensity [55]. When sphere size increased to 10.12 µm, the concentration‐dependent trend remained similar to that observed for the microspheres of 2.87 µm, but the critical concentration for maximum intensity shifted to 3.2 mg/mL (Figure 5H and Figure S21). This can be explained by the fact that, at the same concentration, larger microspheres are fewer in number and spaced farther apart, mitigating light scattering and shielding effects. Thus, a higher concentration is required to reach the balance between scattering and emission enhancement. Similar concentration‐intensity relationship was also observed for TTP@PUM (Figure S22).

The chemical stability of LTP@PUMs in aqueous dispersion was evaluated under different conditions. After 48 h exposure to a Cu^2+^ solution (20 mm) or extreme pH conditions (pH 1 and 14), both ETP@PUM and TTP@PUM maintained their original emission intensity (Figure 5I and Figure S23). This remarkable stability is particularly significant, given that Ln complexes typically undergo fluorescence quenching under such conditions due to displacement of Ln^3+^ by Cu^2+^, formation of Ln hydroxide precipitates in alkaline environment, and protonation of TTA ligands under acidic condition [5, 10, 50, 56]. The observed stability originates from the effective encapsulation and isolation of LTP by PU matrix, shielding Ln^3+^ from these deleterious interactions. Additionally, the microspheres also exhibited good solvent resistance. After 48 h immersion in various organic solvents (n‐hexane, acetone, AN, toluene, THF, DMF, DMSO, chloroform, propylene glycol and pyridine, etc.), no significant change in emission intensity was detected (Figure S24), most likely owing to the inherent solvent resistance of PU matrix [28, 57]. Overall, LTP@PUMs exhibit stable fluorescence in diverse environments, making them promising candidates for various fluorescent applications.

### 2.4 Application of LTP@PUMs in Reusable Detection of 4‐nitrophenol (4‐NP)

**FIGURE 6:**
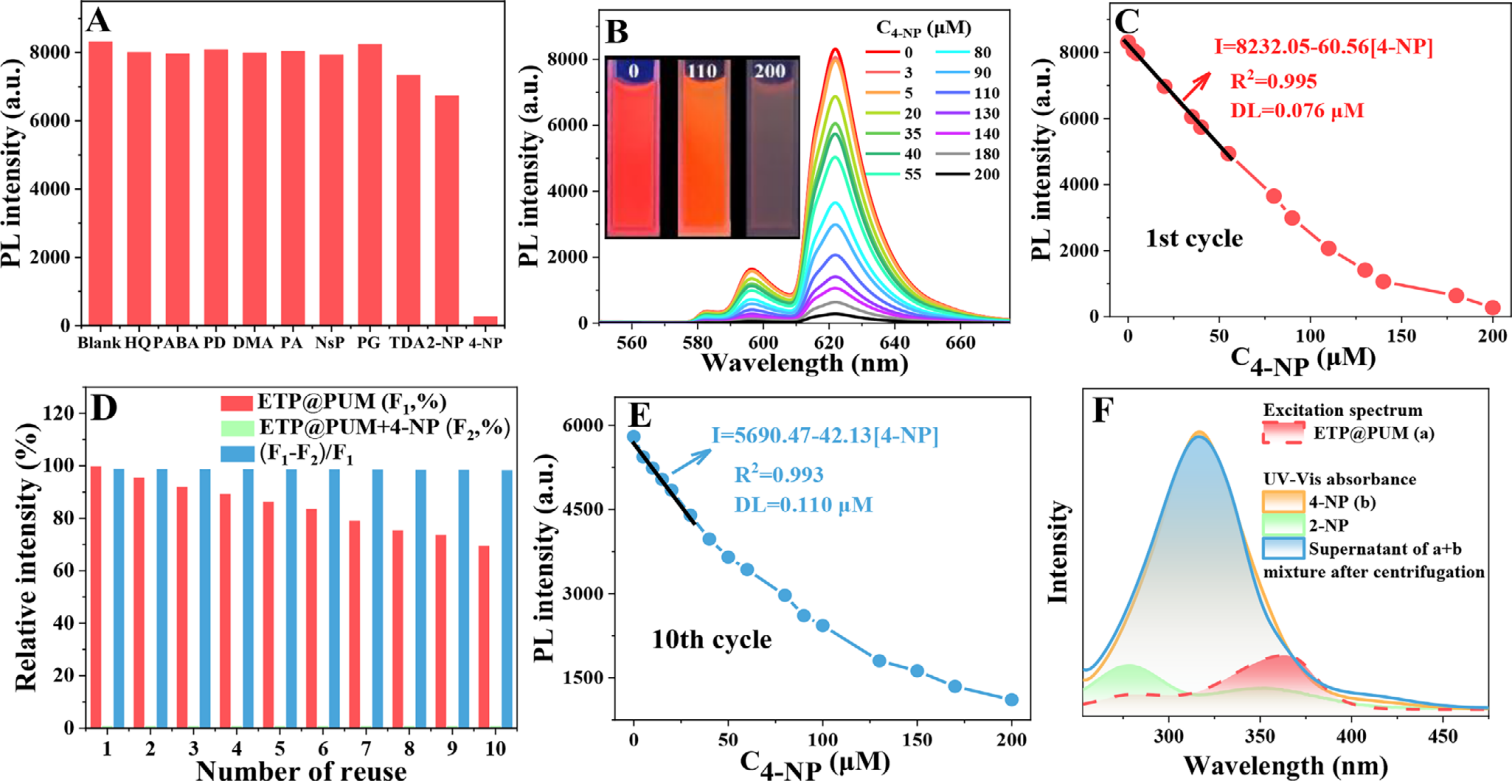
(A) Emission intensity of ETP@PUM dispersion (blank) and upon addition of different aromatic compounds at 200 µM for each; (B) Emission spectra of ETP@PUM with varying [4‐NP]; (C) Emission intensity of ETP@PUM versus [4‐NP] and their linear relationship; (D) Reusability performance: relative emission intensity of ETP@PUM before (F_1_) and after (F_2_) 4‐NP addition, and the quenching efficiency, (F_1_–F_2_)/F_1_, over multiple cycles; (E) Emission intensity and linear relationship for 4‐NP detection using ETP@PUM after 10 reuse cycles; (F) Excitation spectrum of ETP@PUM, UV–vis absorbance of 4‐NP, 2‐NP and the supernatant after mixing ETP@PUM with 4‐NP followed by centrifugation. (ETP@PUM, 4.83 µm, 3.0 mg/mL; λ_ex_ = 350 nm, λ_em_ = 622 nm; analytic concentrations at 200 µM).

LTP@PUM was used as fluorescent sensors for the detection of water pollutants. The emission was measured for an ETP@PUM dispersion (4.83 µm, 3.0 mg/mL) in the presence of 10 different aromatic compounds (HQ, DMA, PG, PA, PABA, p‐PD, TDA, NsP, 2‐NP, 4‐NP), each at a concentration of 200 µm and pH 7.0 under 𝜆_ex_ = 350 nm. As shown in Figure 6A and Figure S25, significant fluorescence quenching was detected only for 4‐NP, while none or slight variation in emission intensity was detected for the rest of the compounds, demonstrating the high quenching selectivity of 4‐NP for ETP@PUM emission. This quenching was then tested at different 4‐NP concentrations ([4‐NP], 0–200 µm, Figure 6B), which shows clearly that the emission intensity was gradually decreasing with increasing [4‐NP] and a quasi‐full quenching was seen at 200 µM. A good linear relationship, between intensity and [4‐NP], was observed in the range of 0–55 µm, and fitted by the equation I = 8232.05−60.56 × [4‐NP] (R^2^ = 0.995, Figure 6C). Using the detection limit (DL) formula DL = 3σ/k [55, 58, 59], where σ represents the standard deviation of the blank signal (1.54) and k the calibration slope (60.56), the DL was determined to be 7.6 × 10^−8^ mol/L (0.076 µm), which is lower than the USEPA limit for [4‐NP] in drinking water (0.43 µm) [55, 59], and better than most of the reported sensors (Table S6), making ETP@PUM a sensor of high sensitivity for 4‐NP detection. For comparison, the same test was then conducted for 4‐NP detection, under the same conditions, using other microspheres (PUM, TTP@PUM, and ETP&TTP@PUM). It is to note that PUM is unsuitable for detection because of its extremely low emission, whereas TTP@PUM and the co‐doped ETP&TTP@PUM exhibited both selective fluorescent quenching by 4‐NP (Figures S26 and S27). As [4‐NP] increased, the emission intensity of both Tb^3+^ (550 nm) and Eu^3+^ (622 nm) significantly decreased. Furthermore, TTP@PUM emission was found to have a good linear response within [4‐NP] of 0–40 µm, giving a DL of 0.39 µm (Figure S26E), as processed above for ETP@PUM. For ETP&TTP@PUM, both Tb^3+^ and Eu^3+^ signals exhibited linear correlations within 0–50 µm, yielding DLs of 0.37 and 0.90 µm, respectively (Figure S27E,F). The higher DL for Eu^3+^ in ETP&TTP@PUM is likely due to its lower content in the microspheres.

Taking ETP@PUM as an example, its reusability in 4‐NP detection was demonstrated here (Figure 6D). After each detection cycle, the microspheres were separated from the dispersion via centrifugation or filtration, washed, and then reused for the next cycle. Although the emission intensity of ETP@PUM decreased by about 30% after 10 reuse cycles, the quenching efficiency by 4‐NP remained above 98.5% ((F_1_‐F_2_)/F_1_, Figure 6D). The calibration curve was reconstructed using ETP@PUM recovered after 10 reuse cycles, yielding a DL of 0.110 µm (Figure 6E). This value, though obviously higher than that in the initial use (0.076 µm, Figure 6C), still outperforms the USEPA standard (0.43 µm) and the reported DLs in a number of sensors (Table S6), demonstrating that ETP@PUM retains reliable detection capability and high sensitivity after multiple uses. In contrast to conventional fluorescent sensing materials (Table S6), which often need to be dissolved in a good solvent and thus are difficult to recover, LTP@PUM presents significant advantages for sustainable environmental sensing due to their high selectivity, sensitivity, and reusability.

The fluorescence quenching mechanism of 4‐NP toward LTP@PUM was then studied, by comparing the UV–vis absorbance of 4‐NP solution (200 µm) with the fluorescence excitation spectrum of ETP@PUM dispersion (3.0 mg/mL, λ_em_ = 622 nm, Figure 6F). It was observed that the excitation spectrum was almost completely overlapped by the absorption spectrum. This is consistent with the inner filter effect (IFE), originated from spectral competition rather than direct molecular interactions [55, 60, 61, 62]. Due to this light competition, a substantial portion of the excitation light is absorbed by 4‐NP in the system, leading to an insufficient excitation of ETP@PUM and thus a fluorescence attenuation by consequence. In contrast, 2‐NP, an isomer of 4‐NP, exhibits a weaker UV–vis absorbance and minimal spectral overlap with the excitation spectrum, resulting in a lower IFE with a modest reduction in emission intensity (Figure 6A,F). Additionally, the UV–vis absorbance of the supernatant, obtained by removing ETP@PUM (3 mg/mL) from its mixture with 4‐NP, closely matches that of pure 4‐NP solution (200 µm, Figure 6F), indicating negligible adsorption of 4‐NP onto ETP@PUM. This result rules out the quenching by surface adsorption or associated intermolecular interactions. The possibility of FRET effect as the dominant mechanism was also excluded. Based on the concentration (3.0 mg/mL) of ETP@PUM dispersion, the average inter‐microsphere distance was estimated to be about 22 µm [63]. With 4‐NP molecules uniformly distributed in the solution, this distance between ETP in ETP@PUM and 4‐NP far exceeds the effective FRET range (<10 nm) [61, 62], making FRET negligible at such macroscopic distances. A strong spectral overlap was also observed between the absorbance of 4‐NP and the excitation spectrum of TTP@PUM (Figure S28), confirming that IFE mechanism applies to different LTP@PUM materials in this study, rather than FRET or direct molecular interactions.

### 2.5 Application of LTP@PUMs in 3D Printing

Among all the 3D printing techniques developed up to date, fused deposition modeling (FDM) is one of the most widely used for fabricating plastic parts owing to its low cost, high speed, simplicity and environmental friendliness [64, 65]. However, the scope for material functionalization via FDM, particularly the incorporation of fluorescence, remains limited, with processing temperatures typically constrained below 200°C [64, 65, 66, 67, 68]. To address this limitation, the performance of LTP@PUMs is evaluated as a fluorescent filler for 3D printing at high temperature. A polycarbonate (PC) matrix, known for its high thermal resistance, was melt‐blended with ETP@PUM using a twin‐screw extruder for compounding and pelletization, followed by filament extrusion via a single‐screw extruder. The processing temperatures mentioned below correspond to the maximum values applied. Results demonstrate that after two extrusion cycles at 260°C, stable fluorescence emission was well retained in the ETP@PUM/PC (3/97) filament (Figure S29), indicating the integrity of fluorescent centers (ETP). This observation is consistent with the images of the filament cross‐section observed under SEM, which show well‐preserved ETP@PUM morphology at 260°C (Figure S30). A significant reduction in emission intensity was observed at 270°C accompanied by a darkening of the filament, and the fluorescence was completely quenched when the temperature reached 275°C (Figure S29). SEM images show that ETP@PUM in the filament maintained their size at 275°C, with however small pores on the surface (Figure S30), likely attributable to thermal degradation of ETP complex. This result indicates that LTP@PUM is of good stability for up to 260°C.

**FIGURE 7:**
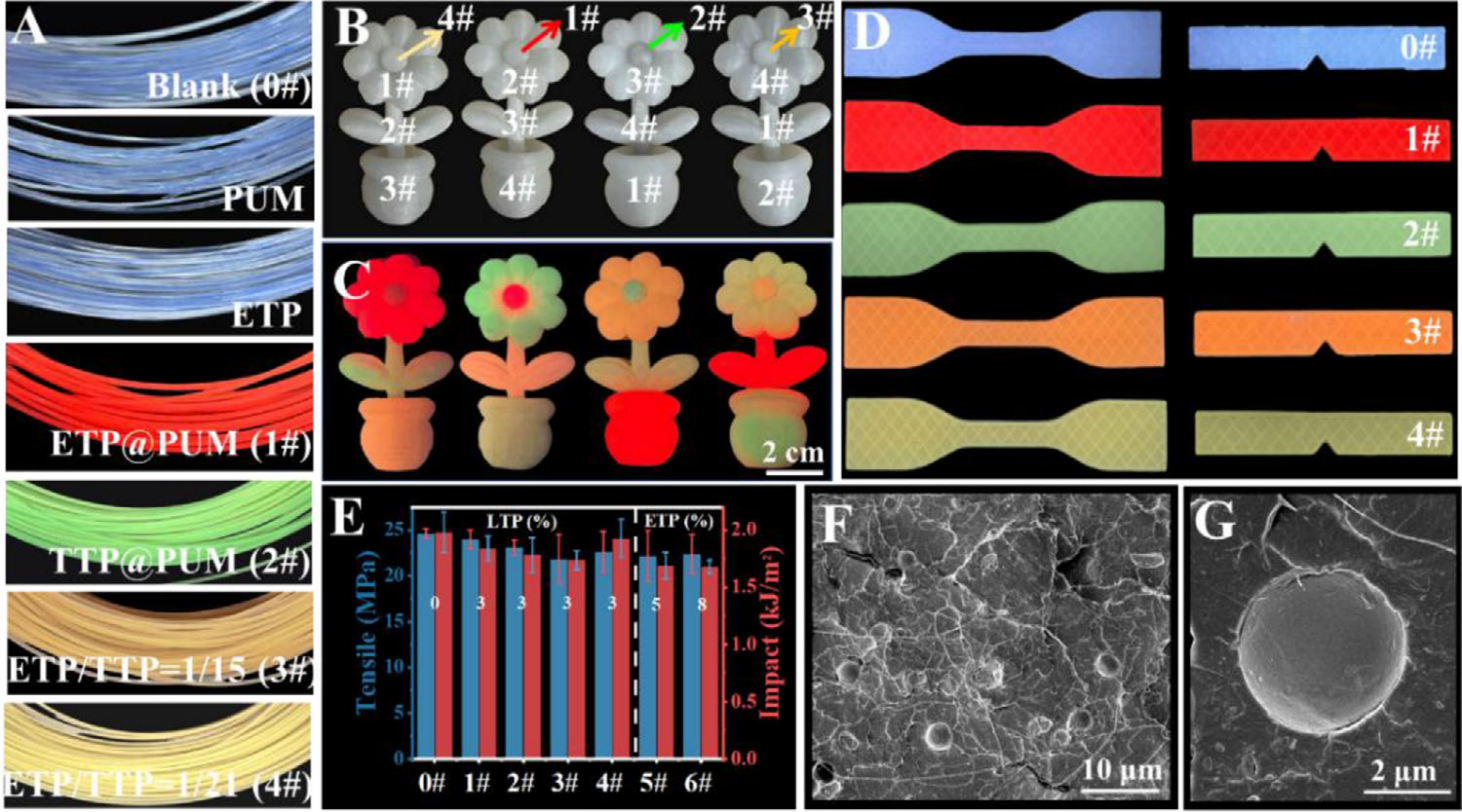
(A) Photographs of PLA and its blends with ETP, PUM and LTP@PUMs after extrusion into filaments; (B, C) Images of a flower model fabricated via four‐channel 3D printing using the obtained filaments under (B) daylight and (C) 365 nm UV light; (D) Photographs of 3D‐printed tensile and impact testing specimens under UV light, and (E) their corresponding impact and tensile strengths; (F, G) SEM images of the fracture surface of impact specimen (1#) at (F) 500× and (G) 5000× magnification.

To assess the 3D printing performance of LTP@PUM, widely used polylactic acid (PLA) in FDM [64, 65, 69] was employed as the matrix. For comparison, control samples containing pure ETP (0.015%) and PUM were also prepared alongside four LTP@PUMs (3.0% loading, equivalent to ∼0.015% pure LTP) of different colors. All mixtures were processed into pellets at 190°C, drawn into filaments at 200°C, and finally fabricated by FDM 3D printing at 220°C. As shown in Figure 7A, pure PLA (sample 0#) and PUM/PLA filaments exhibit only weak blue fluorescence under 365 nm UV light. At the same ETP content (∼0.015%), the ETP@PUM/PLA filament (sample 1#) displays bright red emission, whereas ETP/PLA shows no red fluorescence, likely due to thermal degradation of ETP complex during processing. This contrast demonstrates again that ETP incorporation in PU effectively protects ETP from degradation, and endows them with good fluorescence efficiency at high temperature. Filaments done with TTP@PUM (sample 2#) and ETP&TTP@PUM (3#, ETP/TTP ratio of 1/15; 4#, 1/21) exhibited green, orange, and yellow colors under UV light (Figure 7A), consistent with those of corresponding microspheres (Figure 4). Using a four‐channel 3D printer and the multicolored filaments obtained, a flower model, featuring petals, stamens, stem/leaves, and a flowerpot, was integrally fabricated in a single FDM step. Under daylight, the model is uniformly white (Figure 7B), while under UV light, it displays well‐defined multicolor emissions determined by the color of microspheres used in each component (Figure 7C), showcasing the color programmability of the printing process.

The influence of LTP@PUM incorporation on the mechanical properties of the composites was also evaluated. Dumbbell‐shaped and notched specimens were fabricated via FDM printing using PLA incorporated with different LTP@PUMs, and subjected to mechanical testing (Figure 7D,E). The resulting specimens also exhibited colors in accord with those of the microspheres (Figure 7D). The tensile and impact strengths were, respectively, 24.6 MPa and 1.98 kJ/m^2^ for pure PLA (0#). By adding 3% of LTP@PUMs (1–4#), a slight reduction was detected for the two strength values: with tensile strength ranged between 21.8 and 24.0 MPa, and impact strength between 1.67 and 1.93 kJ/m^2^. Even at higher ETP@PUM loadings of 5% and 8% (5# and 6#), no marked deterioration was observed for these mechanical properties (Figure 7E). SEM images of the fracture surface of specimen 1# (Figure 7F,G) revealed that the microspheres were uniformly dispersed in PLA matrix and maintained their structural integrity and size after processing. The homogeneous distribution of LTP@PUMs is the key factor for achieving the fluorescence and stable mechanical properties. Leveraging the excellent thermal stability and fluorescent efficiency of LTP@PUMs, these results demonstrate the feasibility of 3D printing for bright and programmable fluorescent materials at high temperature (260°C) with an ultralow LTP content of 0.015%, providing thus a simple and promising approach for advanced applications, especially in biomedical field [69].

### 2.6 Application of LTP@PUMs in Latent Fingerprint (LFP) Visualization

**FIGURE 8:**
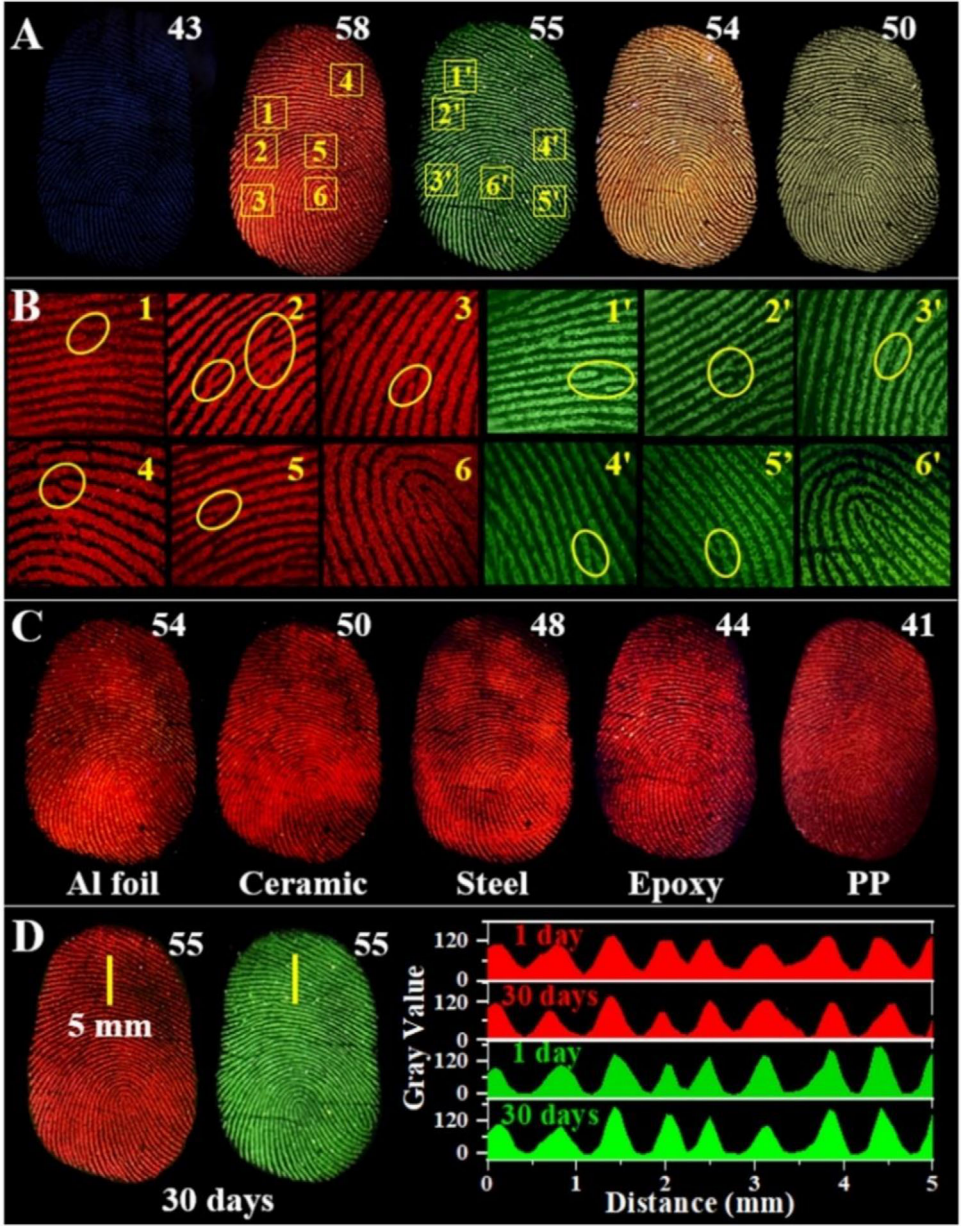
(A) Photographs of LFPs developed using different powder formulations: red LFPs with 1.5% of ETP@PUM; other colors (blue, green, orange and yellow) with 50% of the corresponding microspheres (PUM, TTP@PUM and ETP&TTP@PUMs), with the rest being MMT; (B) High‐magnification images of red and green LFPs on glass substrates; (C) Imaging effects of ETP@PUM‐based LFP powder on different substrates; (D) 30‐day‐aged fluorescent LFPs, along with grayscale values extracted from selected paths (yellow lines) using ImageJ. All images were captured under 365 nm UV light, and numbers in the upper‐right corners were the image quality scores obtained by NFIQ2 method.

To explore the potential application of LTP@PUMs in LFP visualization, fluorescent LFP dry powder was made by directly mixing LTP@PUM microspheres with organic montmorillonite (MMT) using a ball mill. The powder was then gently applied to volunteer fingerprint samples on glass substrate. After removing the powder in excess, the LFP thus made was examined and imaged under 365 nm UV light (Figure 8). For the PUM‐containing powder, a weak fluorescence was seen, with low LFP brightness even at 50% PUM content (Figure 8A). In contrast, a bright red fluorescence was obtained for the LFP made with only 1.5% of ETP@PUM, due to its high QY (75.3%). And distinct green, orange, and yellow LFP images were successfully achieved at an elevated content of 50% (Figure 8A) of the respective composite microspheres, despite their lower QYs (5%–10% for PUM, TTP@PUM, and ETP&TTP@PUM). LFP image was processed through grayscale conversion (Figure S31) and enhancement, and image quality was then evaluated using the standard NFIQ2 algorithm [70, 71, 72, 73], which assigns a score between 0 and 100 based on visual clarity. Scores ≥ 45 are classified as perfect, those between 35 and 44 as good, and those < 6 indicate invalid or poor quality [72, 73]. The resulting quality scores are displayed in the upper‐right corner of each LFP image in Figure 8. PUM‐based powder achieved a score of 43 (good). Although this powder exhibited quite weak fluorescence (Figure 8A), it still produced grayscale images with satisfactory contrast and clarity (Figure S31), resulting in good image quality. A score above 45 (perfect) was obtained for all the rest of LTP@PUM‐based powders, demonstrating their superior performance. High‐magnification imaging revealed that LTP@PUM‐based LFP powders clearly exhibited morphological details, such as ridge patterns, bifurcations, endings and cores (see circular labels in Figure 8B), demonstrating the high resolution and excellent detail retention.

To further assess its general applicability, ETP@PUM‐based LFP powder was applied also to other substrate surface, including Al foil, ceramic tile, steel plate, epoxy painting and polypropylene (PP) plastic (Figure 8C). In addition to the above tested glass substrate, the powders also yielded NFIQ2 scores above 45 (perfect) on Al foil, ceramic tile and steel plate. On epoxy and PP surfaces, the scores fell within the range of 35–45, reflecting a good classification. These relatively lower values can be attributed to the properties of the MMT used (Nanocor I.44P), which is organically modified and has higher affinity for such polymeric substrates, resulting in non‐specific adsorption even in fingerprint valleys devoid of residue (see LFP on PP in Figure 8C). Such background adsorption reduces ridge‐valley contrast, leading to a decrease in the quantitative score.

The imaging durability of the LFP powders was also evaluated on glass substrates. Grayscale images were generated from both freshly prepared and 30‐day‐aged fluorescent LFP, and grayscale values along selected paths (yellow lines in Figure 8D) were extracted using ImageJ software. The results indicated that the image quality retained a perfect score of 55 for both red and green LFPs (Figure 8D), and the grayscale profiles of aged LFPs showed negligible change compared to the initial ones, indicating excellent long‐term imaging stability of these powders. Furthermore, the biosafety of PUM and four types of LTP@PUMs was evaluated through cytotoxicity assays. After 12 h of incubation at concentrations up to 1 mg/mL, all microspheres maintain HeLa cell viability above 96% (Figure S32), which indicates no significant cytotoxicity under the LFP experimental conditions, ensuring operational safety during practical procedures such as fingerprint collection. In summary, the developed LTP@PUM‐based LFP powders exhibit high performance, broad substrate applicability, and excellent biosafety, showing strong potential for forensic use.

### 2.7 In Vivo Metabolic Behavior of LTP@PUMs in Mice

Based on the in vitro cytotoxicity tests (Figure S32), which show LTP@PUMs are of very low toxicity or nontoxicity, they were employed as model particles for PU microplastics (MPs). Their in vivo biodistribution, retention and metabolic behavior were subsequently investigated in a mouse model. To determine whether microsphere aggregation occurs in vivo, we collected blood from the mouse heart at 10 min and 6 h after tail vein injection and examined blood smears under a microscope. At 10 min post injection (Figure S33), numerous microspheres were observed as single, dispersed particles without aggregation. These results confirm that the 10.12 µm microspheres do not aggregate in the bloodstream. Additionally, the overall microsphere content in the mouse blood at 6 h post‐injection was reduced compared to that at 10 min post‐injection (Figure S34), suggesting that the microspheres gradually accumulated in peripheral tissues over time. To further investigate their tissue distributions, the microspheres, including PUM, ETP@PUM and TTP@PUM (2.87 µm), were administered via tail vein injection (30 mg/kg, 100 µL volume). The mice were sacrificed 24 h post‐injection, and major organs were harvested for analysis.

**FIGURE 9:**
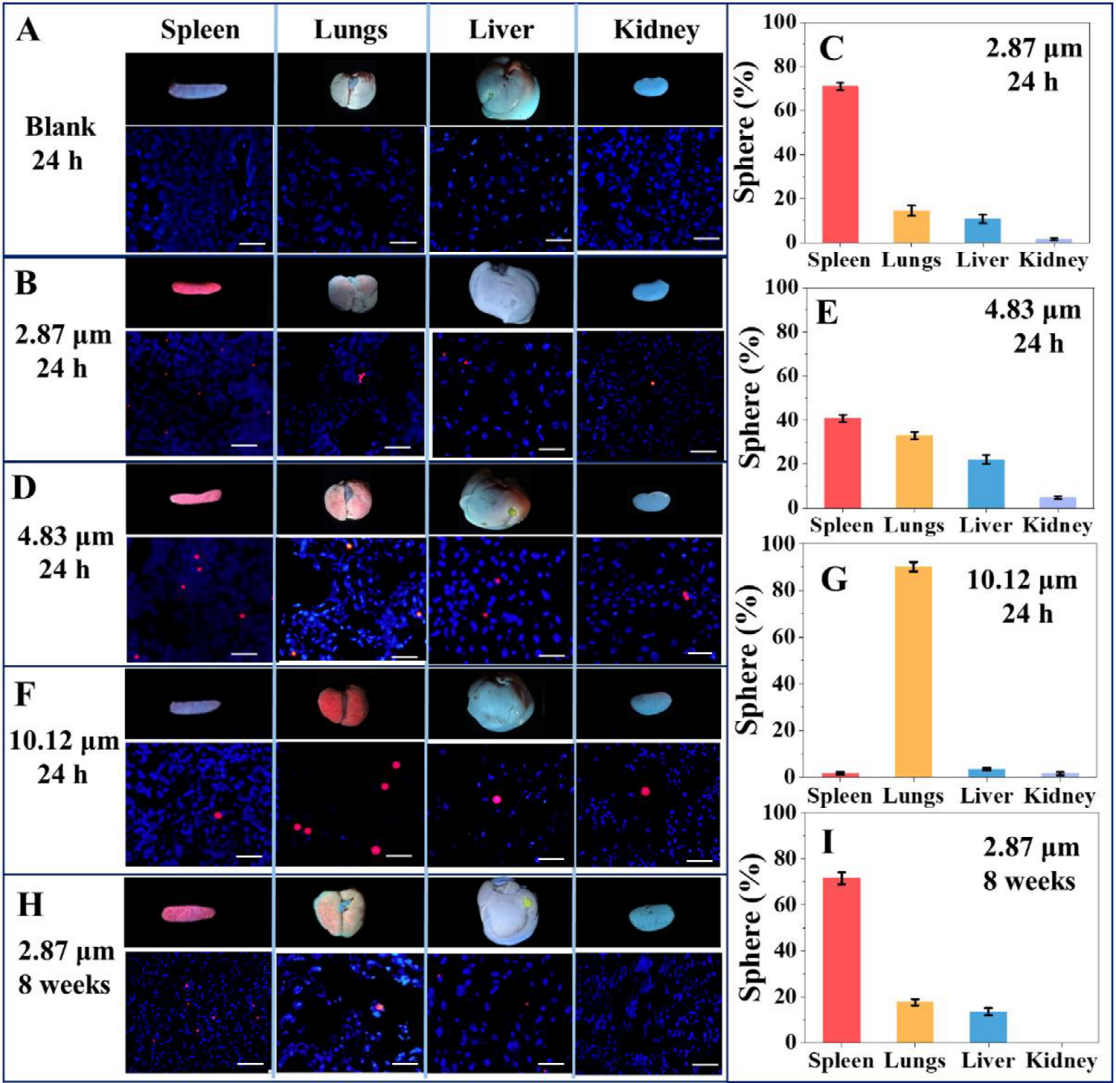
Biodistribution and persistence of ETP@PUM of varying sizes in mice. (A) a blank control mouse after 24 h: photographs under 365 nm UV light and corresponding FMs of DAPI‐stained organ cryosections. (B, C) Mice administered 2.87 µm ETP@PUM and analyzed at 24 h: (B) organ images under UV light and representative FMs; (C) quantitative analysis of the relative proportion of microspheres in each organ, as determined from FMs. (D, E) Mice administered 4.83 µm ETP@PUM at 24 h: (D) organ images and FMs; (E) quantitative analysis of sphere distribution. (F, G) Mice administered 10.12 µm ETP@PUM at 24 h: (F) organ images and FMs; (G) quantitative analysis of sphere distribution. (H, I) Mice administered 2.87 µm ETP@PUM at 8 weeks: (H) organ images and FMs; (I) quantitative analysis of sphere proportion. Data for additional time points (6 h, 1 week, 4 weeks, and 8 months) are provided in Figure S37.

Under 365 nm UV light, strong red fluorescence was observed predominantly in the spleens of the mice injected with ETP@PUM, and weaker signals in the lung and liver. No significant fluorescence was observed in the kidney, heart or ovary (Figure S35A). The presence of ETP@PUM was further confirmed in frozen tissue sections of the spleen, liver and lung by FM following DAPI staining, while only sparse distribution was noted in kidney sections (Figure 9). No red microspheres were found in heart or ovarian tissues (Figure S35B). In contrast, no fluorescence was observed in tissues using PUM or TTP@PUM (Figure S35). These results demonstrate the superior in vivo imaging effect of ETP@PUM. This advantage arises primarily from the high QY (75.3%) of ETP@PUM and emission in the red region (622 nm), where tissue fluorescence is low and spectral overlap with DAPI's blue emission is minimal, thereby avoiding signal crosstalk. The green‐fluorescent TTP@PUM exhibits weaker emission (Figure 5) than ETP@PUM, and is easily masked by DAPI's signal, complicating detection, while non‐fluorescent PUM cannot be optically tracked.

The percentage of ETP@PUM (2.87 µm) in each organ relative to the total recovered amount was then determined (Figure 9C). The results indicated that the majority (about 71.0%) of the microspheres were accumulated in the spleen, followed by the lung (14.6%) and liver (10.8%), with negligible distribution in the other organs tested. This quantitative result is consistent with organ brightness under UV light and FM observations (Figure 9B). The organ‐selective distribution may be explained by size‐matching with the capillary systems of the filtration organs [74, 75, 76, 77, 78]. As primary filtration organs, the lung, liver and spleen possess capillary beds that mechanically capture circulating microspheres. Following intravenous injection, ETP@PUMs enter the heart via the inferior vena cava and initially pass through the pulmonary circulation, making the lungs the initial filtration barrier [74, 75]. Given that the average diameter of mice pulmonary capillaries is about 4.0–4.4 µm [79], microspheres larger than 5 µm will be typically retained there [74, 76, 77]. Since ETP@PUM used here has a diameter of 2.87 µm, smaller than the pulmonary capillaries, only a small fraction (14.6%) is retained in the lung, likely due to incidental lodging. The spleen is capable of effectively capturing microspheres larger than 200 nm [77]. ETP@PUM (2.87 µm) significantly exceeds the splenic filtration threshold, and therefore predominantly retained in the spleen (71.0%) via mechanical filtration during the first circulatory pass. It is likely that the 2.87 µm microspheres are retained at the interendothelial slits or within the splenic sinuses, rather than entering the splenic cords (200‐500 nm), due to a significant size mismatch and the rigid, non‐deformable nature of the PUM material [30]. In contrast, the liver, a major component of the mononuclear phagocyte system, clears particles smaller than 5 µm through phagocytosis by its abundant Kupffer cells and macrophages [76, 77, 78, 80]. The preferential splenic accumulation (71.0% vs 10.8% in the liver) suggests the dominance of mechanical filtration over phagocytic clearance for ETP@PUM. This organ distribution differs from some earlier reports [74, 75, 76], potentially due to the good biocompatibility of LTP@PUMs. It is plausible that the high biocompatibility of ETP@PUM may attenuate the phagocytic response of Kupffer cells and macrophages, and reduce their recognition by the phagocyte system, therefore shifting the primary clearance mechanism from hepatic phagocytosis [74, 75, 76] to splenic mechanical filtration. Organs such as the heart, kidney, and ovary do not function as primary filtration organs and lack active phagocytic mechanisms for particle clearance. Their vascular endothelium possesses tight junctions (about 10–20 nm) [75], which pose a formidable physical barrier to the extravasation of micrometer‐scale particles, thereby preventing ETP@PUM from extravasating and accumulating in these tissues (Figure S35B). The biodistribution of ETP@PUM (2.87 µm) was further investigated in a murine model bearing subcutaneous HeLa tumor xenografts (Figure S36). At 24 h post‐injection, the organs distribution profile remained largely consistent with that in healthy mice, while no microsphere was detected within the tumor tissue (Figure S36B). The size of ETP@PUM (2.87 µm) substantially exceeds the endothelial gaps in tumor vasculature (∼500 nm) [75], which precludes efficient extravasation.

To confirm the size‐matching mechanism for in vivo biodistribution of ETP@PUM, three different diameters (2.87, 4.83, and 10.12 µm) were evaluated (Figure 9B–G). ETP@PUM with a diameter of 2.87 µm accumulated predominantly in the spleen (71.0%), with a combined 25.4% in the lungs and liver (Figure 9B,C). As the microsphere size increased, a decrease in splenic fluorescence intensity was observed (Figure 9D,F). Microsphere proportion in the spleen dropped to 40.7% for the microspheres of 4.83 µm and was nearly abolished at 1.6% for those of 10.12 µm (Figure 9E,G). This was concurrent with a dramatic shift in distribution toward the lung, where the retention proportion rose sharply to 32.9% and 90.1% for the microspheres of size of 4.83 and 10.12 µm, respectively, along with obviously red brightness enhancement (Figure 9D,G), indicating substantial accumulation of larger ETP@PUM in the lung. FM analysis revealed that the microspheres of 10.12 µm retained within pulmonary capillaries, with infiltrating neutrophils or macrophages notably absent. Furthermore, no fibrous encapsulation was observed surrounding the microspheres (Figure 9F), suggesting that they were primarily retained within the lumens of pulmonary capillaries, particularly within the septal microvessels of alveolar regions, without eliciting significant acute inflammatory responses. This size‐dependent retention pattern was paralleled in the liver, where microsphere accumulation correlated inversely with their size, measuring 22.1% for 4.83 µm group compared to only 3.4% for 10.12 µm one (Figure 9E,G). This distribution profile aligns well with size‐matching mechanism [74, 75, 76, 77, 78]. The pulmonary circulation acts as the first filtration barrier, trapping microspheres larger than 5 µm. Accordingly, microspheres with a size of 4.83 µm fell within a “transitional range”: a portion of the microspheres passed through the pulmonary circulation into the systemic circulation and were predominantly captured by the liver and spleen, while the rest was still retained in the lung (Figure 9E), resulting in considerable distribution in the pulmonary tissue. As the size further increased to 10.12 µm, the microspheres were efficiently filtered by lung (Figure 9G), greatly reducing the sphere proportion entering the downstream circulation and, correspondingly, decreasing their distribution to liver and spleen. The specific accumulation of ETP@PUMs in spleen (2.87 µm) and lung (10.12 µm) highlights their potential as carriers for targeted drug delivery.

The retention of 2.87 µm ETP@PUM in various mouse organs was evaluated at different time points after injection (Figure S37). Results indicated that the emission intensity and sphere proportion in spleen, liver, and lung remained largely stable. Even at 8 months post‐injection, no significant decrease in sphere proportion was detected in those organs (Figure S37G,H), demonstrating long‐term persistence without effective clearance. SEM analysis of these organ sections was employed to assess the in vivo stability of the microspheres (Figure S38). Results revealed that ETP@PUM maintained their original morphology and size in organs without noticeable alteration 8 weeks after. In addition, SEM analysis also verified the presence and accumulation of both non‐fluorescent PUM and TTP@PUM in various organs, which also maintained their structural integrity over the 8‐week period (Figure S38). Although the biodistribution of PUM and TTP@PUM could not be tracked via fluorescence imaging, their confirmed presence and stability, suggest that they exhibit comparable in vivo metabolic behavior. Furthermore, all mice administered microspheres (PUM, ETP@PUM, and TTP@PUM) exhibited no obvious abnormal behavior and survived the entire eight‐month observation period, indicating favorable biosafety at the organism level. Specifically, no abnormalities were observed in general condition parameters, including locomotor activity, hair luster, and body weight (Figure S39A,B). Analysis of harvested organs showed that microspheres persisted in the spleen for 8 months, with red fluorescence still detectable under UV irradiation, indicating stable long‐term retention (Figure S37G). Moreover, serum liver biochemical parameters in microsphere‐treated mice remained within the normal range and showed no significant differences from controls, further confirming that even prolonged in vivo retention of microspheres does not cause obvious adverse effects (Figure S39C–J). Obviously, the long‐term accumulation of these microspheres in specific organs suggests potential risks such as physical blockage or chronic inflammation. However, the fluorescent traceability, organ selectivity and long‐term stability of ETP@PUM make them promising candidates for applications, such as biotracer or targeted drug carrier.

## 3 Conclusion

This work presents a facile one‐step precipitation polymerization for the preparation of LTP‐doped polyurea microspheres (LTP@PUMs) under mild, additive‐free, and quiescent conditions. The method features operational simplicity, benign reaction parameters, and low LTP loading (0.5%), significantly reducing material cost and process complexity. The size (2–10 µm) and fluorescence color (red, orange, yellow, green) of the microspheres are easily adjustable with good control, by modulating reaction temperature, LTP complex type and ETP/TTP ratios. The resulting LTP@PUMs exhibited a high fluorescence QY up to 75.3%, a highly superior emission efficiency. Moreover, they maintained stable fluorescence after extended 365 nm irradiation, 80°C thermal treatment (48 h), and exposure to harsh chemical environments, including strong acids (pH 1), alkalis (pH 14), Cu^2+^ solutions, and solvents of varying polarity, indicating their remarkable photostability and chemical robustness. These properties are attributed to the strong HB and coordination interactions within LTP@PUMs, as well as the favorable compatibility between LTP and PU matrix, which were all demonstrated through FTIR, XPS, TGA, DSC, and EDS analyses. Owing to the excellent redispersibility and high QY, LTP@PUMs were successfully applied as fluorescent sensors for sensitive detection of 4‐NP, achieving a DL of 0.076 µm and maintaining high sensitivity over 10 reuse cycles. Leveraging their redispersibility and biocompatibility, LTP@PUMs were combined with MMT for LFP identification, yielding high‐quality images with a broad substrate adaptability and good durability. LTP@PUMs also sustained their fluorescent properties after high‐temperature processing at 260°C, enabling their use as thermally stable multicolor fillers in FDM 3D printing, a distinctive feature rarely reported. In the biomedical field, in vivo biodistribution study revealed a pronounced size‐dependent organ selectivity of LTP@PUMs, where microspheres of the size 2.87 µm preferentially accumulated in spleen (71.0%), and large microspheres of the size 10.12 µm were efficiently trapped in lung (90.1%), without inducing obvious abnormal behaviors over an eight‐month period. This size‐governed biodistribution provides important insights into the safety and biological fate of PU‐based MPs and highlights the potential of LTP@PUMs in bio‐tracking and targeted drug delivery. This work therefore highlights the multifunctional applications of LTP@PUMs across several advanced fields, including environmental monitoring, public security, advanced manufacturing and biomedicine.

## 4 Experimental Section

This section, including seven subsections (Materials, Synthesis of TTP, Preparation of PUM and LTP@PUMs, Materials Processing and 3D Printing, Preparation and Evaluation of LTP@PUM/MMT composites for LFP Imaging, Cytotoxicity test, In Vivo Evaluation of LTP@PUMs in Mice, and Characterizations), is placed in the Supporting Information.

## Funding

This work is financially supported by Natural Science Foundation of Shandong Province (ZR2023QH521, ZR2025MS147, ZR2025LZL009), National Natural Science Foundation of China (82401944, 22375028), Taishan Scholars Young Experts Program (tsqn202306389), and Collaborative Academic Innovation Project of Shandong Cancer Hospital (FC015).

## Conflicts of Interest

The authors declare no conflicts of interest.

## Supporting information




**Supporting File**: advs76091‐sup‐0001‐SuppMat.docx.

## Data Availability

The data that support the findings of this study are available from the corresponding author upon reasonable request.
