# Supplementary material for: One‐Step Preparation of Lanthanide‐Doped Polyurea Multicolor Microspheres for Versatile Fluorescent Applications
Source: Adv Sci (Weinh). 2026 Jun 12;13(50):e76091. doi: 10.1002/advs.76091 (PMC13336787; doi:10.1002/advs.76091)
Supplement: Supplementary file 1 [file ADVS-13-e76091-s001.docx]

**Supporting Information**

**One-Step Preparation of Lanthanide-Doped Polyurea Multicolor Microspheres for Versatile Fluorescent Applications**

*Guiyu Zhang, Xiaoxia Yu, Xinhui Wang, Xiang Zheng Kong, Xiangling Gu, Jitao Liu, Shusheng Li, Xiaoli Zhu, Lianbao Cao^*^, and Xubao Jiang^*^*

**1. Experimental Section**

*1.1. Materials*

Isophorone diisocyanate (IPDI), *N,Nˊ*-dimethylformamide (DMF), dimethyl sulfoxide (DMSO), hydroquinone (HQ), p-aminobenzoic acid (PABA), *N,Nˊ*-dimethylaniline (DMA), p-phenylenediamine (PD), phenylamine (PA), phloroglucinol (PG), 2,4-toluenediamine (TDA), 4-nitrophenol (4-NP), 2-nitrophenol (2-NP), 4-nitrobenzaldehyde (NBA), 4-nitrosophenol (NsP), pyridine, organic montmorillonite (MMT, Nanocor I.44P), CuSO_4_·5H_2_O, EuCl_3_·6H_2_O, Tb(NO_3_)_3_·6H_2_O, 1,10-phenanthroline monohydrate (Phen·H_2_O), 2-thiophenyl trifluoroacetone (TTA) and Eu(TTA)_3_Phen (ETP) were all analytical pure (AP) grade and purchased from Shanghai Aladdin Biochemical Technology Co. Ltd. Acetonitrile (AN), methanol, tetrahydrofuran (THF), chloroform, acetone, n-hexane, toluene, propylene glycol, NaOH, all AP grade, were obtained from Tianjin Fuyu Fine Chemical Co. Ltd. Concentrated HCl (AP Grade) was purchased from Yantai Yuandong Fine Chemical Co. Ltd. KBr, spectrum pure, was from Sinopharm Chemical Reagent Co. Ltd. Polylactic acid (PLA) was supplied by Zhejiang Hisun Biomaterials Co., Ltd. Polycarbonate (PC‑110) was obtained from Chi Mei Corporation. HeLa cells (CCL2) were purchased from Huankai Microbial Sci. & Tech. Co. Ltd. DMEM/F12 with L-glutamine & 15 mM HEPES, penicillin-streptomycin (100×) and trypsin-EDTA (0.25%, phenol red-free) were acquired from M&C Gene Technology. Fetal bovine serum (FBS) was from ExCell Bio. The CCK‑8 Cell Counting Kit was provided by Vazyme. PFA fixative solution (4%) was obtained from Biosharp. PBS and 4′,6‑diamidino‑2‑phenylindole (DAPI) were sourced from Servicebio. OCT compound was obtained from Thermo Fisher Scientific. Double distilled water was lab-made ultra-pure (Flom, FFX1002-Up, Qingdao, China).

*1.2. S*ynthesis *of Tb(TTA)_3_Phen (TTP)*

In a typical process,^[1]^ Tb(NO_3_)_3_·6H_2_O (1.3869 g) and TTA (2.0403 g) were dissolved in methanol (90 mL) in a flask equipped with a magnetic stirrer. The solution was stirred at 60 °C for 0.5 h, after which the pH was adjusted to 8.0 using a saturated NaOH methanol solution. The reaction was continued for another 0.5 h before adding Phen·H_2_O (0.6068 g). Stirring was maintained for 1 h following precipitate formation. The final product TTP was obtained by filtration, methanol washing and drying at 70 °C for 12 h.

*1.3. Preparation of PUM and LTP@PUMs*

*1.3.1. Preparation of PUM*

PUM were prepared via precipitation polymerization using IPDI as the sole monomer in a H_2_O/AN mixed solvent with mass ratio at 20/80, following a previously reported procedure.^[2,3]^ In a typical procedure, a 120 mL screw-cap glass bottle was charged with the H_2_O/AN mixture (92.0 g), followed by the addition of IPDI (8.0 g). The container was sealed, manually shaken to achieve a homogeneous mixture, and incubated statically (without agitation) in a water bath maintained at 50 °C. The initially transparent solution, composed of IPDI, H_2_O and AN, turned turbid after about 109 min, and then the reaction was allowed to proceed for additional 3 h. The resulting microspheres were isolated from the reaction medium by centrifugation, washed three times with H_2_O/AN, and dried under vacuum at 90 °C for 4 h.

*1.3.2. Preparation of LTP@PUMs*

The preparation of LTP@PUMs followed a similar procedure to that used for pure PUM, with the key modification being the addition of LTP (0.04 g) along with IPDI (8.0 g) into the solvent prior to polymerization. Subsequent steps involving polymerization and post-treatment were carried out in an identical manner. A series of composite microspheres were fabricated by varying the type of LTP complex (ETP, TTP, and their mixtures), LTP amount (relative to IPDI), ETP/TTP mass ratio, IPDI loading and polymerization temperature. Based on the type of LTP used, the resulting microspheres were correspondingly designated as ETP@PUM, TTP@PUM and ETP&TTP@PUM, respectively.

*1.4.* *Materials Processing and 3D Printing of LTP@PUMs containing Composites*

*1.4.1. Thermal Stability Evaluation of LTP@PUM Using PC Matrix.*

To assess the thermal stability of LTP@PUM, its composites were prepared with PC matrix. ETP@PUM (ETP amount, 0.5%) were blended with PC-110 in mass ratios of 3/97 or 6/94. Melt compounding was performed using a twin-screw extruder with the following temperature profile: feeding zone, 240 °C; middle zone, 250-270 °C; discharge zone, 260-280 °C; with a 10 °C gradient maintained between the middle and discharge zones. The extrudate was pelletized, and filaments were produced using a single-screw extruder at a temperature matching the discharge zone of the twin-screwing process.

***1.4.2. Filament Fabrication and Model Printing with PLA Matrix.***

For structural and functional evaluation, LTP@PUMs were blended with PLA. A mixture of LTP@PUM (LTP amount, 0.5%) and PLA with mass ratio of 3/97 was melt-compounded in the twin-screw extruder with temperature set to 110 °C (feeding), 180 °C (middle) and 190 °C (discharging), respectively. The extrudate was pelletized and drawn into uniform filament (1.65-1.75 mm) using a monofilament lineator at 200 °C. Four distinct filaments were prepared using specific colored LTP@PUMs (red, orange, yellow, green) blended with PLA matrix.

***1.4.3. Materials 3D Printing with*** *M****ulti-color Fluorescence***

The resulting filament was used in a four-channel 3D printer via FDM at 220°C to fabricate different specimens, including standard mechanical testing samples, floral and mouse models. As a key demonstration, a multi-color fluorescent flower model was fabricated through the separate loading of four distinct filaments into the printer channels, achieving complex structural integrity with region-specific fluorescence.

*1.5. Preparation and Evaluation of LTP@PUM/MMT composites for LFP Imaging*

*1.5.1. Preparation of LTP@PUM/MMT Composites*

The fluorescent composites based on LTP@PUM and MMT were prepared through mechanical ball milling. Specifically, LTP@PUM and MMT were mixed in designated proportions and processed in a ball mill for 1 h. Five types of fluorescent powders were prepared, with the red-emitting one containing 1.5% ETP@PUM, while the blue (PUM), green (TTP@PUM), orange and yellow (ETP&TTP@PUM, ETP/TTP=1/15 and 1/21) ones contained 50% microspheres with the remainder consisting of organic MMT.

*1.5.2. Preparation of LFP Samples*

Prior to sampling, volunteers washed their hands with distilled water and air-dried naturally. The fingertip was then gently rubbed against the forehead or both sides of the nose to simulate the natural secretion of a mixture of sweat and skin lipids. The fingertip was then pressed with uniform pressure (about 2-5 s) onto clean surfaces of various substrates, including glass, Al foil, ceramic tile, epoxy-coated surfaces and PP, to deposit invisible LFPs.

*1.5.3. Imaging of LFP*

The as-prepared powders were evenly applied over the LFPs on different substrates using a powder dispenser. Excess powder was gently removed with a stream of air. After a brief settling period (<10 s), the developed fingerprints were illuminated under 365 nm UV light and imaged using a smartphone camera (Apple iPhone 15 Pro Max, 4032×3024 pixels).

*1.5.4. Quality Evaluation of LFP Images*

The quality of the developed LFP images was evaluated through grayscale analysis and quantitative image scoring. Grayscale conversion was performed using ImageJ software (National Institutes of Health, USA), and grayscale profiles along defined paths were extracted to assess contrast. Image quality was further evaluated using the NIST Fingerprint Image Quality (NFIQ 2), which are formally standardized as part of ISO/IEC 29794-4 and serve as the reference implementation of the standard.^[4,5]^

*1.6. Cytotoxicity test of LTP@PUMs*

HeLa cells were seeded in 96-well plates at a density of 4000 cells per well (counted using a hemocytometer) and cultured for 24 h under standard conditions (37 °C, 5% CO_2_). After adherence, cells were treated with LTP@PUM suspensions in DMEM at final concentrations of 0 (control), 50, 100, 500, and 1000 mg/L (n=6 per group). Following 12 h of incubation, cells were gently washed three times with ice-cold PBS. Cell viability was then assessed using a CCK-8 assay, wherein 100 μL of CCK-8/culture medium mixture (1/9) was added to each well. After 1 h of incubation, the absorbance at 450 nm was measured with a microplate reader. Cell viability was calculated as (OD_sample−OD_blank)/(OD_control−OD_blank). All experiments were performed in triplicate. Statistical significance was determined by one-way ANOVA using Origin software, with p<0.05 considered significant.

*1.7. In Vivo Evaluation of LTP@PUMs in Mice*

*1.7.1. Ethical Statement and Experimental Animals*

All animal procedures were conducted in accordance with protocols approved by the Animal Ethics Committee of Cancer Hospital of Shandong First Medical University (Approval No. SDTHEC202604012). Two mouse models were used: female C57BL/6J mice (6 weeks old, ~20 g) for evaluating the biodistribution and clearance of microspheres in immunocompetent hosts, and female BALB/c nude mice (4-6 weeks old) for establishing subcutaneous xenograft tumors to mimic the tumor microenvironment. All mice were housed under specific pathogen-free (SPF) conditions with a 12 h light/dark cycle, at 22-24 °C, with free access to food and water. Bedding was changed weekly to maintain sanitary conditions.

*1.7.2. Establishment of Subcutaneous Tumor Model*

To investigate microsphere distribution in tumor tissue, a subcutaneous xenograft model was established. Briefly, HeLa cells were resuspended in 1× PBS and inoculated into the right flank of nude mice at a density of 5×10^6^ cells in a 100 μL volume. Experiments were initiated when tumor volumes reached about 100-300 mm^3^.

*1.7.3. In Vivo Administration and Experimental Design*

For in vivo administration, microspheres were dispersed in sterile saline to a concentration of 6 mg/mL via vortex mixing and sonication. Female C57BL/6J mice received a single tail vein injections of 100 μL suspensions at a dose of 30 mg/kg. Tissues were harvested 24 h post-injection to compare the in vivo imaging performance of differently colored microspheres, including blue PUM, red ETP@PUM and green TTP@PUM. Subsequent experiments examined the effects of ETP@PUM of three diameters (2.87, 4.83 and 10.12 μm; 24 h post-injection) and administration duration (6 h to 8 weeks, 2.87 μm) on in vivo metabolism; In tumor-bearing nude mice, tissues were collected 24 h after administration. At predetermined time points, mice were euthanized, and major organs (heart, liver, spleen, lungs, kidneys, ovaries) and tumor tissues were collected for analysis. Each experimental group included n=3 mice, with a control group receiving an equal volume of sterile saline.

*1.7.4. Fluorescence Imaging and Histological Analysis*

Harvested tissues were embedded in OCT compound, flash-frozen in liquid nitrogen, and sectioned into 5 μm thick slices using a cryostat. Sections were fixed with 4% PFA, and nuclei were stained with DAPI. Fluorescence images were acquired using a FM, and the distribution and retention of LTP@PUMs were analyzed based on their intrinsic fluorescence signals. The quantitative analysis of ETP@PUM distribution in each organ was conducted by calculating the number of microspheres per unit tissue volume from the sectional area (determined by FM) and a slice thickness of 5 μm. The percentage of microspheres per organ relative to the total recovered was then calculated using organ mass and reference density.^[6]^ Statistical analysis was determined by one-way ANOVA with p<0.05.

*1.7.5. Serum Biochemical Analysis*

Serum samples were obtained by collecting whole blood via orbital bleeding after anesthesia with tribromoethanol (300 μL of 1.25% solution per mouse), allowed to clot at room temperature for 2 h, and then centrifuged at 4 °C and 3,000 rpm for 15 min. Biochemical parameters were then analyzed using an automated clinical chemistry analyzer (Servicebio, GH-100). The levels of alanine aminotransferase (ALT) and aspartate aminotransferase (AST) were determined by the kinetic (Rate) method at 340 nm. Total bilirubin (TBIL) and direct bilirubin (DBIL) were measured using the vanillylacetamide oxidation endpoint method at 450 nm. Albumin (ALB) was quantified by the bromocresol green endpoint method at 630 nm. Alkaline phosphatase (ALP), gamma-glutamyl transferase (γ-GT), and total bile acid (TBA) were assayed by kinetic methods at 405 nm. All assays were performed using standard commercial reagent kits.

*1.8. Characterizations*

Morphologies of PUM and LTP@PUMs were observed under scanning electronic microscope (SEM, Quanta FEG-250, FEI). By counting at least 200 spheres, number-average size (D_n_), weight-average size (D_w_) and size distribution (D_w_/D_n_) were obtained.^[2,3]^ Eu and Tb contents were measured by inductive coupled plasma optical emission spectrometer (ICP-OES, Agilent 5800). Fourier Transform Infrared analysis (FTIR) was done on a Spectrum GX of PerkinElmer with the samples compressed in KBr pellet. X-ray photoelectron spectroscopy (XPS) was performed on an AXIS Supra instrument (Shimadzu). Thermogravimetric analysis (TGA) and differential scanning calorimetry (DSC) were conducted on TGA 55 and DSC 25 (TA instruments), respectively, under N_2_ protection with a heating rate of 10 °C/min. Crystal phase was checked on an X-ray diffractometer (XRD, D8 Focus, Brüker). Energy-dispersion spectroscopy (EDS) was used to obtain element mapping with an Oxford instrument (X-Max^N^ 50). Fluorescence emission was recorded on an F-7000 spectrometer (Hitachi) with a slit width of 2.5/2.5 nm (excitation/emission) for solid samples and 5.0/2.5 nm for aqueous suspensions. The absolute quantum yield (QY) and lifetime (τ) were measured using a steady/transient-state fluorescence spectrometer (FLS920, Edinburgh) equipped with an integrating BaSO_4_ sphere as the reference. UV absorption was tested using a UV-Vis spectrophotometer (Lambda 35, PerkinElmer). A vertical planetary ball mill (JZ-22(23)-XQM, Hebi Jingzhong Technology) was employed to blend microspheres with MMT. Melt blending and pelletizing of microsphere-resin composites were conducted using a mini twin-screw extruder (SJZS-10B) coupled with an air-cooled conveyor (SJSF-120, Wuhan Ruiming Instrument). For 3D printing filament fabrication, an ARTME 3D desktop extruder (MK3S+) was used, and FDM printing was performed on a Bambu Lab P1S system. The tensile strength was conducted on an Instron 5569 testing machine with a crosshead speed of 50 mm/min at room temperature. Impact strength was measured using an MTS tester (ZBC8400-B) with a pendulum energy of 4.0 J. Microsphere suspensions (100 μL per injection) were administered via tail vein in mice using a YLS-Q9G injector (Jinan Yiyan Technology). Tissue sections were prepared with a CryoStar NX50 cryostat, and cell cultures were maintained in a Forma^TM^ 31 series CO_2_ incubator (Thermo Scientific). Optical density for cell viability were obtained with a SpectraMax® ABS Plus microplate reader (Molecular Devices). Microsphere and tissue section were observed using an Olympus IX73 inverted fluorescence microscope (FM, Olympus).

**2. Characterization and Results**

**Figure S1.** Chemical structure of TTA, Phen and LTP

**Figure S2.** SEM pictures of ETP@PUMs obtained at different polymerization time (A: 98 min; B: 106 min; C: 114 min; D: 180 min)

**Table S1.** Effect of ETP amount (based on IPDI added) on ETP@PUM prepared at 50 °C with 8% IPDI

| ETP amount (%) | Turbidity time (min) | Sphere yield (wt%) | ETP content (mg/g) | ETP loading rate (%) | D_n_ (μm) | D_w_/D_n_ |
| --- | --- | --- | --- | --- | --- | --- |
| 0 | 109 | 88.95 | 0 | 0 | 7.13 | 1.010 |
| 0.20 | 103 | 90.21 | 2.26 | 90.21 | 5.21 | 1.016 |
| 0.30 | 99 | 90.82 | 3.25 | 87.17 | 5.12 | 1.016 |
| 0.40 | 98 | 91.62 | 4.26 | 86.54 | 4.96 | 1.012 |
| 0.50 | 95 | 93.92 | 5.12 | 85.40 | 4.83 | 1.008 |
| 0.60 | 94 | 94.32 | 5.33 | 74.49 | 4.76 | 1.009 |
| 0.70 | 90 | 94.66 | 5.17 | 62.22 | 4.73 | 1.056 |

**Figure S3.** SEM pictures of ETP@PUMs prepared at 50 °C with 8% IPDI and different ETP amount (A, 0.3%; B, 0.6%; C, 0.7%)

**Table S2.** Effect of IPDI loading on ETP@PUMs prepared at 50 °C with 0.5% ETP (based on IPDI added)

| IPDI loading (%) | Turbidity time (min) | Sphere yield (wt%) | ETP content (mg/g) | ETP loading rate (%) | D_n_ (μm) | D_w_/D_n_ |
| --- | --- | --- | --- | --- | --- | --- |
| 4.0 | 104 | 93.38 | 5.19 | 86.07 | 5.47 | 1.048 |
| 8.0 | 95 | 93.92 | 5.12 | 85.40 | 4.83 | 1.008 |
| 12.0 | 92 | 93.54 | 4.97 | 82.57 | 5.13 | 1.007 |
| 16.0 | 85 | 94.92 | 4.89 | 82.43 | 6.18 | 1.012 |
| 17.0 | 83 | 95.16 | 4.74 | 80.11 | 7.21 | 1.241 |

**Figure S4.** SEM pictures of ETP@PUMs prepared at 50 °C with 0.5% ETP and different IPDI loading (A, 12%; B, 16%; C, 17%)

**Table S3.** Effect of polymerization temperature on ETP@PUMs prepared with 8.0% IPDI and 0.5% ETP

| Temperature  (℃) | Turbidity time (min) | Sphere yield (wt%) | ETP content (mg/g) | ETP loading rate (%) | D_n_ (μm) | D_w_/D_n_ |
| --- | --- | --- | --- | --- | --- | --- |
| 30 | 148 | 94.52 | 5.25 | 88.13 | 10.12 | 1.016 |
| 50 | 95 | 93.92 | 5.12 | 85.40 | 4.83 | 1.008 |
| 70 | 88 | 92.25 | 4.99 | 81.75 | 2.87 | 1.009 |

**Table S4.** Effect of TTP amount (based on IPDI) on TTP@PUMs prepared at 50 °C with 8% IPDI

| TTP amount (%) | Turbidity time (min) | Sphere yield (wt%) | TTP content (mg/g) | TTP loading rate (%) | D_n_ (μm) | D_w_/D_n_ |
| --- | --- | --- | --- | --- | --- | --- |
| 0 | 109 | 88.95 | 0 | 0 | 7.13 | 1.005 |
| 0.2 | 105 | 90.14 | 2.28 | 90.94 | 4.97 | 1.016 |
| 0.3 | 102 | 90.89 | 3.37 | 90.46 | 4.94 | 1.012 |
| 0.4 | 99 | 91.87 | 4.25 | 86.58 | 4.82 | 1.014 |
| 0.5 | 96 | 93.43 | 5.20 | 86.28 | 4.81 | 1.008 |
| 0.6 | 93 | 94.28 | 5.36 | 74.87 | 4.83 | 1.009 |
| 0.7 | 91 | 94.54 | 5.19 | 62.38 | 4.79 | 1.022 |

**Figure S5.** SEM pictures of TTP@PUMs prepared at 50 °C with 8% IPDI and different TTP amount (A, 0.3%; B, 0.6%; C, 0.7%)

**Table S5**. Effect of ETP/TTP ratio (total amount fixed at 0.5% of IPDI added) on ETP&TTP@PUMs prepared at 50 °C with 8% IPDI

| ETP/TTP mass ratio | Turbidity time (min) | Sphere yield (wt%) | ETP | | TTP | | D_n_ (μm) | D_w_/D_n_ |
| --- | --- | --- | --- | --- | --- | --- | --- | --- |
|  |  |  | Content (mg/g) | Loading rate (%) | Content (mg/g) | Loading rate (%) |  |  |
| 1/13 | 86 | 92.67 | 0.35 | 80.22 | 4.89 | 86.64 | 4.96 | 1.012 |
| 1/15 | 88 | 92.88 | 0.31 | 81.39 | 4.92 | 86.53 | 4.97 | 1.006 |
| 1/17 | 94 | 92.32 | 0.29 | 85.13 | 5.03 | 87.29 | 5.08 | 1.014 |
| 1/19 | 94 | 92.32 | 0.26 | 84.80 | 5.00 | 86.27 | 5.04 | 1.013 |
| 1/21 | 95 | 91.39 | 0.24 | 85.24 | 5.04 | 85.67 | 5.09 | 1.008 |
| 1/25 | 96 | 90.57 | 0.21 | 87.35 | 5.03 | 84.12 | 5.13 | 1.017 |

**Figure S6.** FTIR spectra of TTA, Phen, ETP, TTP (A), IPDI, PUM, and LTP@PUMs (B)

**Figure S7.** Survey (A) and deconvolved (B, Eu^3+^; C, Tb^3+^) XPS spectra of LTP@PUMs

**Figure S8.** Survey (A) and deconvolved (B, C 1s; C N 1s; D, O 1s; E, Eu^3+^; F, Tb^3+^) XPS spectra of TTP@PUM and ETP&TTP@PUM

**Figure S9.** DSC (A) and TGA (B) curves of TTP@PUM and ETP&TTP@PUM

**Figure S10.** XRD diffractogram of PUM, ETP@PUM, TTP@PUM and ETP&TTP@PUM

**Figure S11**. EDS elemental mappings of TTP@PUM

**Figure S12**. Emission spectra of ETP, TTP (A) and PUM (B) under different excitation wavelength

**Figure S13**. Emission spectra of ETP@PUM with different ETP amount under different excitation wavelength (IPDI loading, 8.0%)

**Figure S14**. Emission spectra of ETP@PUMs prepared with 0.5 % ETP and different IPDI concentration (A, 4%; B, 8%; C, 12%; D, 16%)

**Figure S15**. Emission spectra of ETP@PUMs prepared with 0.5% ETP and 8.0% IPDI at different polymerization temperature (A, 30℃; B, 50℃; C, 70℃)

**Figure S16**. Emission spectra of TTP@PUMs prepared with 8.0% IPDI and different TTP amount under different excitation wavelength

**Figure S17**. Emission spectra of ETP&TTP@PUMs prepared with 8.0% IPDI and different ETP/TTP mass ratio under different excitation wavelength (total amount of ETP+TTP, 0.5%)

**Figure S18**. (A) UV-Vis absorbance of PUM and excitation spectra of ETP and EuCl_3_ (λ_em_=622 nm); (B, C) Luminescence decay curves of pure PUM (B: 𝜆_ex_=360 nm, 𝜆_em_=400 nm) and Tb^3+^ (C: 𝜆_ex_=360 nm, 𝜆_em_=550 nm) emission in TTP@PUMs prepared with different TTP amount.

**Figure S19**. Fluorescence intensity of Eu^3+^ and Tb^3+^ in LTP@PUMs after heating at 85 °C for different durations (λₑₓ=360 nm; λₑₘ=622 nm for Eu^3+^, λₑₘ=550 nm for Tb^3+^).

**Figure S20**. Fluorescence emission spectra of ETP@PUM (A) and TTP@PUM (B) aqueous dispersions (3 mg/mL) at different excitation wavelength, and their emission intensities after standing at room temperature for different time periods (C, ETP@PUM; D, TTP@PUM; λₑₓ=350 nm, λₑₘ=622 nm for Eu^3+^, λₑₘ=550 nm for Tb^3+^).

**Figure S21.** Fluorescence intensity (λₑₓ=350 nm, λₑₘ=622 nm) and transmittance (measured at 525 nm) of ETP@PUMs aqueous dispersions with different sphere sizes (A, 2.87 μm; B, 10.12 μm) at various concentrations.

**Figure S22.** Fluorescence intensity and transmittance of TTP@PUMs aqueous dispersion at different concentrations (λₑₓ=350 nm, λₑₘ=550 nm).

**Figure S23.** Fluorescence intensity of TTP@PUM aqueous dispersion after immersion in aqueous solutions at pH = 1 and  14 for 48 h (λₑₓ=350 nm, λₑₘ=550 nm).

**Figure S24.** Fluorescence intensity of ETP@PUM and TTP@PUM after immersion in different solvents for 48 h (λₑₓ=350 nm, λₑₘ=622 nm for Eu^3+^, λₑₘ=550 nm for Tb^3+^).

**Figure S25.** Emission spectra of ETP@PUM dispersions upon addition of different pollutants (λ_ex_=350 nm).

**Table S6.** DL comparison of this work with reported results for 4-NP detection

| Material | DL (μM ) | Linear region (μM) | Ref. |
| --- | --- | --- | --- |
| CdTe QDs/CTAB probe | 18.0 | 25~300 | [7] |
| MoS2 nanosheets | 7.0 | 10~1500 | [8] |
| N-doped porous carbon | 5.44 | 2~400 | [9] |
| Bismuth film electrode | 3.40 | 1~100 | [10] |
| CeO_2_-Cu_2_O/CH nanocomposites | 2.03 | 74~375 | [11] |
| Cr-MOF modified carbon electrode | 0.70 | 2~500 | [12] |
| Ag-doped ZnOnanostructures | 0.67 | 0.67~9 | [13] |
| SnO_2_@ZIF-8/gC_3_N_4_ nanohybrids | 0.57 | 100~100 | [14] |
| N-doped carbon dots | 0.40 | 1~250 | [15] |
| ZnCo_2_O_4_ nanosheets | 0.30 | 1~4000 | [16] |
| Zn-MOF | 0.28 | 30~200 | [17] |
| Fluorescent polymer carbon dots | 0.26 | 0.5~60 | [18] |
| Electrochemical sensor based on MIP | 0.20 | 2~400 | [19] |
| CPU microspheres | 0.15 | 0.15~40 | [20] |
| Cucurbit[8]uril-basedsupramolecular | 0.15 | 4~30 | [21] |
| Pd-graphene composite and PNIPAM | 0.10 | 0.5~250 | [22] |
| TTP@PUM | 0.39 | 0~40 | This work |
| ETP@PUM | 0.076 | 0~55 |  |

**Figure S26.** (A) Emission spectra of TTP@PUM dispersions after adding different pollutants, and (B) corresponding emission intensity at 550 nm; (C) emission spectra of TTP@PUMs in the presence of 4-NP of different concentration, and (D) corresponding emission intensity at 550 nm; (E) linear relationship between emission intensity and [4-NP] (0~40 μM) and the corresponding DL (λ_ex_=350 nm).

**Figure S27.** (A) Emission spectra of the ETP&TTP@PUMs dispersion after adding different pollutants, and (B) corresponding emission intensities at 550 nm (Tb^3+^) and 622 nm (Eu^3+^); (C) emission spectra of ETP&TTP@PUMs at different [4-NP], and (D) corresponding emission intensities at 550 nm (Tb^3+^) and 622 nm (Eu^3+^); (E, F) Linear relationships between emission intensity at 550 nm (Tb^3+^, E) and 622 nm (Eu^3+^, F) versus [4-NP] (0~50 μM), and the corresponding DLs (λ_ex_=350 nm).

**Figure S28.** UV-Vis absorbance of 2-NP and 4-NP aqueous solutions (200 μM), and fluorescence excitation spectrum of TTP@PUM dispersion (3.0 mg/mL; λem=550 nm).

**Figure S29.** Photographs of filaments (PC and its blends with different amount of ETP@PUM) under 365 nm UV light. Filaments were prepared by melt blending and pelletization using a twin-screw extruder (feed zone at 240 °C, the middle and discharge zones differing by 10 °C), followed by extrusion through a single-screw extruder (at the same temperature as discharge zone of the twin-screw extruder). The temperatures indicated in the figure are those at the extruder’s discharge zone.

**Figure S30**. SEM pictures of the fracture surfaces corresponding to the filaments in Figure S29 (A, 260 ℃ with PC+3.0%ETP@PUM; B, 265 ℃ with PC+3.0%ETP@PUM; C, 275 ℃ with PC+6.0%ETP@PUM).

**Figure S31**. Grayscale images of fluorescent LFPs images developed using different powder formulations as listed in Figure 8A

**Figure S32**. The viability of HeLa cells after 14 h culturing in the presence of PUM and LTP@PUMs of different concentration

**Figure S33.** (A) Flowchart of injecting 10.12 μm microspheres into mice, preparing blood smears 10 min post-injection, and observing them under a fluorescence microscope; Bright-field image of the smear (B), image under UV irradiation (C), fluorescence microscopy image (D), and locally magnified view (E). The numbers in (D) indicate the identified red microspheres.

**Figure S34.** Blood smears prepared 6 h after injecting 10.12 μm microspheres into mice: bright-field image of the smear (A), image under UV irradiation (B), and fluorescence microscopy image of the slide (C). The red dot in the middle image of (C) is a microsphere.

**Figure S35.** Biodistribution of PUM, ETP@PUM and TTP@PUM in mice at 24 h post-injection. (A) Representative ex vivo photographs of major organs from each treatment group under a 365 nm UV light. (B) Corresponding representative fluorescence micrographs (FM) of DAPI-stained cryosections from the organs shown in (A).

**Figure S36.** Biodistribution of ETP@PUM in a HeLa tumor xenograft model. Ex vivo organ images (under 365 nm UV light) and corresponding representative FM of tissue sections from: (A) Blank control tumor-bearing mice; (B) Tumor-bearing mice injected with 2.87 μm ETP@PUM and analyzed 24 h post-injection; (C) Those injected with 10.12 μm ETP@PUM and analyzed 24 h post-injection.

**Figure S37.** Time-dependent biodistribution of 2.87 μm ETP@PUM in mice. (A, C, E, G) Photographs of selected organs under 365nm UV light and corresponding FM of DAPI stained organ cryosections at 6 h (A), 1 week (C), ,4 weeks (E) and 8 months (G) post-injection; (B, D, F, H) Quantitative analysis of ETP@PUM in various organs based on FM at 6 h (B), 1 week (D), 4 weeks (F) and 8 months (H) post-injection.

**Figure S38.** SEM analysis of microsphere morphology in organ sections. (A-C) Spleen section collected 24 h post-injection of ~2.8 μm PUM (A), ETP@PUM (B) and TTP@PUM (C); (D) Lung section after 24 h injection of 10.12 μm ETP@PUM; (E, F) Spleen (E) and lung (F) sections obtained 2 months post-injection of 2.87 μm ETP@PUM.

**Figure S39.** Observation of general status of mice at 8 months following microsphere administration: (A) mouse survival status and (B) body weight changes; (C-J) Serum liver biochemical indicators in mice at 8 months after microsphere injection.

**References Cited in This Supporting Information**

[1] M. L. Di Lorenzo, M. Cocca, G. Gentile, M. Avella, D. Gutierrez, M. Della Pirriera, M. Kennedy, H. Ahmed, J. Doran, *J. Colloid. Interf. Sci.* **2013**, *398*, 95–102.

[2] H. Sun, Y. Wei, X. Z. Kong, X. Jiang, *Polymer* **2021**, *216*, 123432.

[3] X. Jiang, D. Zhang, Y. Wang, R. Wang, X. Z. Kong, X. Zhu, S. Li, X. Gu, *ACS Appl.Mater.Interfaces.* **2024**, *16*, 57672–57686.

[4] Z. H. Pu, J. He, X. Liu, J. Wang, Q. H. Bai, C. H. Wang, X. Xiao, *Sensor Actuat. B Chem.* **2025**, *423*, 136874.

[5] A. Makrushin, V. S. Mannam, J. Dittmann, *Appl. Sci.* **2023**, *13*, 10000.

[6] Tissue Properties: density, Online Available: https://itis.swiss/virtual-population/tissue-properties/database/density/

[7] X. Yan, H. Li, X. Wang, X. Su, *Talanta* **2015,** *131*, 88–94.

[8] L. Chen, L. Ji, J. Zhao, X. Zhang, F. Yang, J. Liu. *J. Electroanal. Chem.* **2017,** *801*, 300–305.

[9] L. Hu, F. Peng, D. Xia, H. He, C. He, Z. Fang, J. Yang, S. Tian, V.K. Sharma, D. Shu, *ACS Sustain. Chem. Eng.* **2018,** *6*, 17391–17401.

[10] R. N. Nuñez, J. M. Betancourth, P. I. Ortiz, V. Pfaffen, *Ind. Eng. Chem. Res.* **2019,** *58*, 12411–12418.

[11] S.B. Khan, K. Akhtar, E.M. Bakhsh, A.M. Asiri, *Appl. Surf. Sci.* **2019,** *492*, 726–735.

[12] C. Hu, P. Pan, H. Huang, H. Liu, *Biosensors* **2022,** *12*, 813.

[13] D. Thakur, A. Sharma, D.S. Rana, N. Thakur, D. Singh, T. Tamulevicius, M. Andrulevicius, S. Tamulevicius, S.K. Shukla, S. Thakur, *Chemosensors* **2020,** *8*, 108.

[14] D. Mohanta, A. Mahanta, S.R. Mishra, S. Jasimuddin, M. Ahmaruzzaman, *Environ. Res.* **2021,** *197*, 111077.

[15] D. Das, R. K. Dutta, *ACS Appl. Nano Mater.* **2021,** *4*, 3444–3454.

[16] J. Zhang, S. Cui, Y. Ding, X. Yang, K. Guo, J.T. Zhao, *Biosens. Bioelectron.* **2018,** *112*, 177–185.

[17] J. Li, Y. Liang, S. Wu, Y. Zhang, M. Zhu, E. Gao, *Inorg. Chem. Commun.* **2022,** *143*, 109724.

[18] L. Han, S. G. Liu, J. Y. Liang, Y. J. Ju, N. B. Li, H. Q. Luo, *J. Hazard. Mater.* **2019,** *362*, 45–52.

[19] S. Ata, M. Feroz, I. Bibi, I. Mohsin, N. Alwadai, M. Iqbal, *Syn. Met.* **2022,** *287*, 117083.

[20] X. Jiang, S. Yan, H. Sun, X. Kong, S. Li, H. Shi. *J. Colloid. Interf. Sci*. **2024,** *673*, 550–563.

[21] J. He, X. Y. Yu, Z. C. Yu, M. Liu, P. H. Shan, C. Redshaw, Y. Huang, Z. Tao, X. Xiao, *Anal. Chim. Acta* **2022,** *1226*, 340262.

[22] D. Xu, R. Li, Q. Wang, Y. Yang, G. Wang, Z. Li, *Microchem. J.* **2022,** *172*, 106970.
